# Homeoviscous Adaptation of the Acinetobacter baumannii Outer Membrane: Alteration of Lipooligosaccharide Structure during Cold Stress

**DOI:** 10.1128/mBio.01295-21

**Published:** 2021-08-24

**Authors:** Carmen M. Herrera, Bradley J. Voss, M. Stephen Trent

**Affiliations:** a Department of Infectious Diseases, College of Veterinary Medicine, University of Georgia, Athens, Georgia, USA; b Department of Microbiology, College of Art and Sciences, University of Georgia, Athens, Georgia, USA; KUMC

**Keywords:** *Acinetobacter*, acylation, acyltransferase, cell envelope, cold shock, lipid A, lipooligosaccharide, lipopolysaccharide, outer membrane

## Abstract

To maintain optimal membrane dynamics, cells from all domains of life must acclimate to various environmental signals in a process referred to as homeoviscous adaptation. Alteration of the lipid composition is critical for maintaining membrane fluidity, permeability of the lipid bilayer, and protein function under diverse conditions. It is well documented, for example, that glycerophospholipid content varies substantially in both Gram-negative and Gram-positive bacteria with changes in growth temperature. However, in the case of Gram-negative bacteria, far less is known concerning structural changes in lipopolysaccharide (LPS) or lipooligosaccharide (LOS) during temperature shifts. LPS/LOS is anchored at the cell surface by the highly conserved lipid A domain and localized in the outer leaflet of the outer membrane. Here, we identified a novel acyltransferase, termed LpxS, involved in the synthesis of the lipid A domain of Acinetobacter baumannii. A. baumannii is a significant, multidrug-resistant, opportunistic pathogen that is particularly difficult to clear from health care settings because of its ability to survive under diverse conditions. LpxS transfers an octanoate (C8:0) fatty acid, the shortest known secondary acyl chain reported to date, replacing a C12:0 fatty acid at the 2′ position of lipid A. Expression of LpxS was highly upregulated under cold conditions and likely increases membrane fluidity. Furthermore, incorporation of a C8:0 acyl chain under cold conditions increased the effectiveness of the outer membrane permeability barrier. LpxS orthologs are found in several Acinetobacter species and may represent a common mechanism for adaptation to cold temperatures in these organisms.

## INTRODUCTION

The outer membrane (OM) of Gram-negative bacteria is an asymmetric bilayer mainly composed of lipopolysaccharides (LPS) or lipooligosaccharides (LOS) in the outer leaflet and glycerophospholipids in the inner leaflet of the membrane (1). The asymmetric nature of the OM provides resistance to antimicrobial agents and various environmental stresses (2). LPS consists of three distinct regions that includes the highly conserved lipid A domain, a core oligosaccharide that is attached to the lipid A membrane anchor, and the outermost O-antigen polysaccharide that extends off the core. In comparison to LPS, LOS is a smaller glycoform that contains an extended core oligosaccharide but lacks O antigen (3). Proper synthesis and transport of LPS/LOS under diverse growth conditions are critical for maintaining the OM permeability barrier.

**FIG 1:**
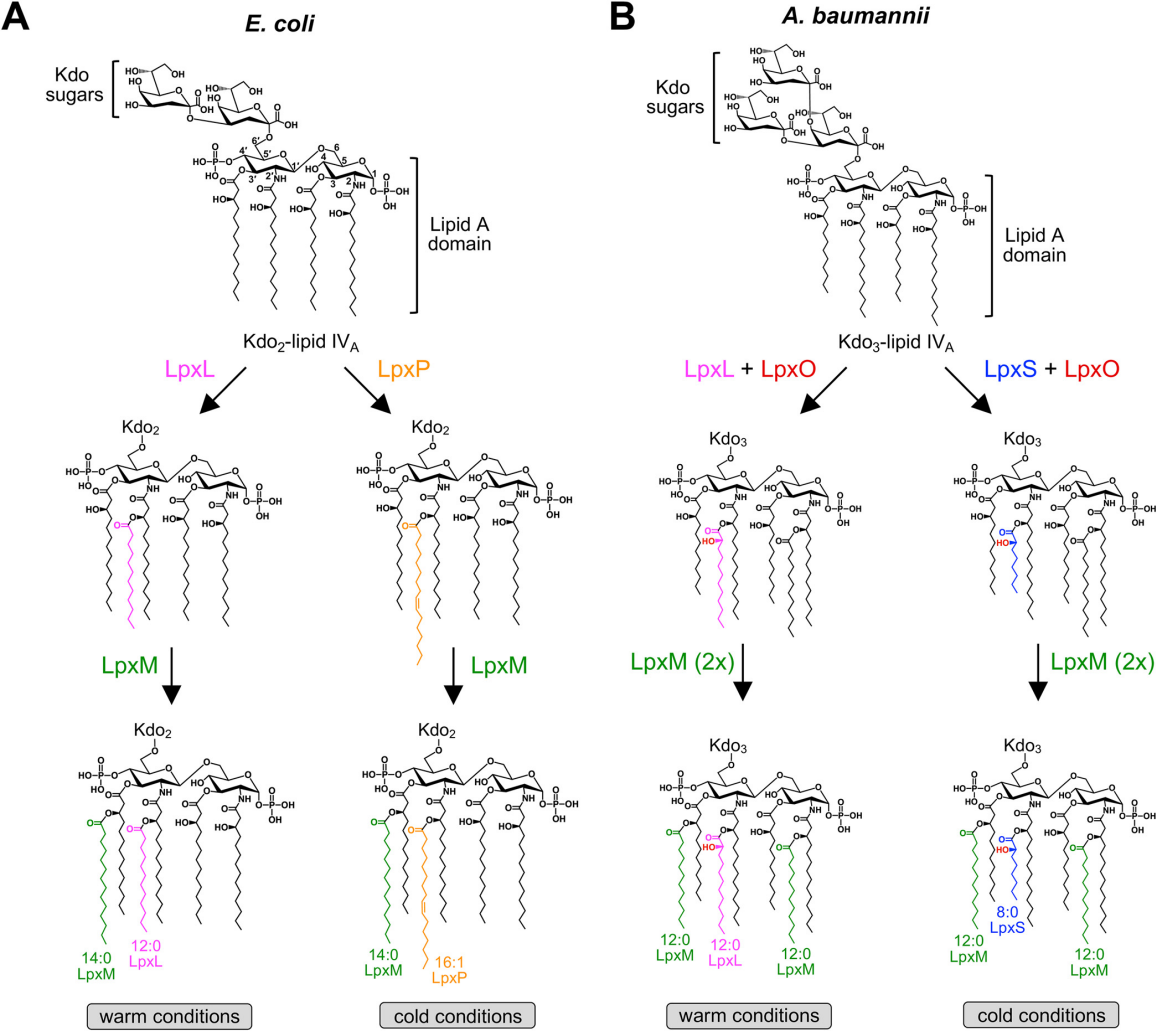
Lipid A secondary acylation steps in E. coli and A. baumannii. (A) The early steps of lipid A biosynthesis lead to the production of a *bis*-phosphorylated, tetra-acylated disaccharide precursor. This is followed by the addition of two 3-deoxy-d-*manno*-octulosonic acid (Kdo) sugars producing Kdo_2_-lipid IV_A_. Although technically part of the core oligosaccharide of LPS, the Kdo sugars must be added to the E. coli lipid A backbone before the final steps of lipid A synthesis can occur. At higher growth temperatures, LpxL then adds a laurate (C12:0) at the 2′ position that is followed by the incorporation of a myristate (C14:0) at the 3′ position by LpxM. However, the acylation pattern changes under cold conditions (12°C) with the replacement of the C12:0 fatty acid with a palmitoleate (C16:1). Under these conditions, LpxP replaces LpxL, altering lipid A structure and outer membrane fluidity. (B) In A. baumannii, 3 Kdo sugars are added to the tetra-acylated disaccharide lipid A precursor, yielding Kdo_3_-lipid IV_A_. Whether or not Kdo addition is necessary for function of the secondary acyltransferases in A. baumannii is unknown. Both LpxL and LpxM of A. baumannii incorporate C12:0 fatty acids; however, LpxM_Ab_ is bifunctional, acylating lipid A at both the 2 and the 3′ positions. Also, LpxL_Ab_ and LpxM_Ab_ do not function in a sequential manner. During growth under cold conditions, A. baumannii induces expression of LpxS that adds an octanoate (C8:0) to the 2′ position, similar to LpxL. A hydroxyl group is added to the secondary chain at the 2′ position by the LpxO dioxygenase, in a chain-length-independent manner. For clarity, secondary acyl chains have been assigned a color along with its corresponding enzyme: magenta (LpxL), green (LpxM), orange (LpxP), and blue (LpxS). Hydroxylation by LpxO is represented by the color red.

In Escherichia coli, lipid A is comprised of a glucosamine disaccharide backbone that is both phosphorylated and fatty acylated (Fig. 1A).

During lipid A biosynthesis, four “primary” β-hydroxyacyl chains are attached to the disaccharide backbone and two additional “secondary” fatty acids are attached to the β-hydroxy groups at the 2′ and 3′ positions. Addition of the first two sugars of the core oligosaccharide, two 3-deoxy-d*-manno*-oct-2-ulosonic acid (Kdo) residues, precedes the secondary acylation steps, and for this reason Kdo addition is required for complete lipid A synthesis. Two inner membrane-bound enzymes, LpxL and LpxM, esterify a laurate (C12:0) and myristate (C14:0) fatty acid to the hydroxy groups of the primary acyl chains at the 2′ and 3′ positions, respectively (4) (Fig. 1A).

Notably, in E. coli, secondary acylation steps are sequentially ordered, whereas in other Gram-negative bacteria (e.g., Helicobacter pylori) this is not the case (5). For this reason, E. coli *lpxL* mutants display a significant growth defect at elevated temperatures (>32°C) (6). Since E. coli LpxM activity is partially dependent upon LpxL, *lpxL* mutants produce predominantly tetra- and penta-acylated lipid A anchors. The growth defect arises from lack of efficient LPS transport by the ABC (ATP binding cassette) transporter MsbA that “flips” LPS from the cytoplasmic to the periplasmic leaflet of the inner membrane. Both *lpxM* and *msbA* can serve as multicopy suppressors of *lpxL* mutants, compensating for the lack of 2′ acylation, and restore LPS transport.

In mesophilic bacteria like E. coli and Salmonella, cold shock at 12°C induces synthesis of the late acyltransferase LpxP, a paralog of LpxL. LpxP transfers a palmitoleate (C16:1), an unsaturated fatty acid, to the 2′ position, increasing membrane fluidity (Fig. 1A) (7, 8). Expression of LpxP restores both hexa-acylated lipid A production in *lpxL* mutants and LPS transport. Much like the changes seen in the glycerophospholipid fraction at lower temperatures, including increases in unsaturated fatty acids or reduction in acyl chain length (9, 10), temperature-regulated changes in the lipid A structure represent an important mechanism of homeoviscous adaptation.

For example, Francisella novicida encodes two temperature-regulated LpxD primary acyltransferases that act early in the lipid A biosynthetic pathway. The LpxD expressed at 18°C transfers a shorter acyl chain, compared to its counterpart expressed at 37°C (11). In Pseudomonas aeruginosa, the levels of laurate and palmitate fatty acids in lipid A decrease while the levels of 2-hydroxylaurate and 3-hydroxydecanoate increase at lower growth temperatures (12). Psychrophilic bacteria also display lipid A remodeling that likely contributes to homeoviscous adaptation. *Psychrobacter* species, facultative psychrophiles, increase the length of LPS acyl chains with increasing growth temperature (13, 14), whereas the obligate psychrophiles Colwellia psychrerythraea and Psychromonas marina (15, 16) maintain both primary and secondary unsaturated acyl chains to support proper outer membrane fluidity.

Thus, despite the fact that lipid A biosynthesis is both highly conserved and essential in nearly all Gram-negative bacteria, variation in acyl chain selectivity and positional specificity of acyltransferases lead to structural differences that can directly influence bacterial fitness. Furthermore, often bacteria have latent enzymes that can drastically alter the lipid A structure following the conserved biosynthetic pathway. These structural changes also influence bacterial fitness by impacting resistance to antimicrobial agents (e.g., antimicrobial peptides), altering recognition by the mammalian innate immune system via the Toll-like receptor-4 (TLR4)/myeloid differentiation factor 2 (MD2) receptor during colonization (17), and allow for maintenance of membrane fluidity under different growth conditions.

Here, we expand on previous work from our laboratory on the assembly and maintenance of the outer membrane of A. baumannii. The genus Acinetobacter is associated with diverse ecological niches and belongs to the family *Moraxellaceae*, which includes genera *Moraxella*, *Psychrobacter*, and others (18). Several Acinetobacter species are important nosocomial opportunistic pathogens. Within this group, A. baumannii has globally emerged as one of the most critical multidrug-resistant pathogens (19, 20). An array of molecular mechanisms for adaptation to harsh surrounding conditions alongside antibiotic resistance contributes to a limited effectiveness of current therapeutic strategies including the so-called “last-resort” cationic antimicrobial peptide antibiotics, the polymyxins (21).

A. baumannii synthesizes LOS, which lacks O antigen, with an extended core oligosaccharide. It produces a mixture of hexa- and hepta-acylated lipid A species with hepta-acylated lipid A as the dominant form (Fig. 1B). Similar to E. coli, A. baumannii LpxL adds an acyl chain at the 2′ position (Fig. 1); however, the fatty acid is hydroxylated by the dioxygenase LpxO (22) (Fig. 1B). LpxO, considered a lipid A modification enzyme, is present in multiple pathogens (e.g., Salmonella) but absent in E. coli. Another key difference from the canonical E. coli pathway is that A. baumannii LpxM is a bifunctional enzyme that adds two fatty acids, at the 2 and 3′ positions, giving rise to hepta-acylated species. Both acyltransferases add C12:0 fatty acids and exhibit site specificity. However, late acylation in A. baumannii is not an ordered process as deletion of either acyltransferase does not impact activity of the second (23). Our previous characterization of the two secondary acyltransferases in A. baumannii found the acylation pattern plays an important role in resistance to key antibiotics and desiccation survival, likely potentiating the bacterium’s ability to persist in diverse settings (23). In this study, we have discovered a third A. baumannii secondary acyltransferase, A1S_1255 (ATCC 17978 designation), which is present in several Acinetobacter species. The protein is an LpxL homologue and transfers a secondary octanoate (C8:0) to the 2′ position (Fig. 1B).

To our knowledge, this is the shortest reported secondary acyl chain present in Gram-negative bacteria LPS or LOS. Accordingly, we refer to A1S_1255 as LpxS, where “S” indicates short. In addition, we determined that *lpxS* is upregulated at low temperature in multiple A. baumannii strains. This mechanism of lipid A alteration likely modulates membrane fluidity and outer membrane permeability at cold temperatures. Furthermore, the octanoylated lipid A variant weakens the TLR4-dependent inflammatory response. Given the usefulness of lipid A variants for modulation of the immune response (e.g., adjuvant design), LpxS will further expand the repertoire of synthetically engineered lipid A molecules for vaccine design.

## RESULTS

### A. baumannii encodes a third possible lipid A late acyltransferase.

Lipid A secondary acyltransferases are encoded by a multigene family and function during the latter steps of lipid A synthesis. These enzymes incorporate secondary acyl chains converting tetra-acylated lipid A precursors into penta-, hexa-, or hepta-acylated species depending upon the organism (Fig. 1) (5, 24, 25). Previously, our group characterized A. baumannii LpxL and LpxM, describing their function in secondary lipid A acylation under optimal growth conditions (23). Searching for late acyltransferase homologues using the Prokaryotic Genome Analysis Tool Database (PGAT) (26), we found the gene *A1S_1255* (now termed *lpxS*) annotated as a putative lauroyl (C12:0) acyltransferase in strain 17978. Amino acid sequences analyzed by Protein Basic Local Alignment Search Tool (BLASTp) determined that ATCC 17978 LpxS shares approximately 99% identity with putative LpxS acyltransferases (E = 0.0), 65% with LpxL (E = 2e−154), and 25% with LpxM (E = 1e−18) homologues within 15 A. baumannii species included in PGAT. Additionally, a search for secondary acyltransferases among Acinetobacter species identified that several genomes have one copy of a predicted LpxS ortholog (see Table S1 and Fig. S1 in the supplemental material). Interestingly, some of the analyzed Acinetobacter species encode between 3 and 5 predicted lipid A acyltransferases (Table S1).

10.1128/mBio.01295-21.1 FIG S1 LpxS lipid A acyltransferase is distributed within Acinetobacter species. Phylogenetic distribution of selected Acinetobacter species is based on 16S rRNA. Acinetobacter strains that contain a single copy of *lpxS* in their genomes are indicated in blue. Download FIG S1, PDF file, 0.3 MB. Copyright © 2021 Herrera et al. 2021 Herrera et al. https://creativecommons.org/licenses/by/4.0/ This content is distributed under the terms of the Creative Commons Attribution 4.0 International license.

10.1128/mBio.01295-21.7 TABLE S1 Distribution of putative lipid A secondary acyltransferases in Acinetobacter. Download Table S1, PDF file, 0.07 MB. Copyright © 2021 Herrera et al. 2021 Herrera et al. https://creativecommons.org/licenses/by/4.0/ This content is distributed under the terms of the Creative Commons Attribution 4.0 International license.

**FIG 2:**
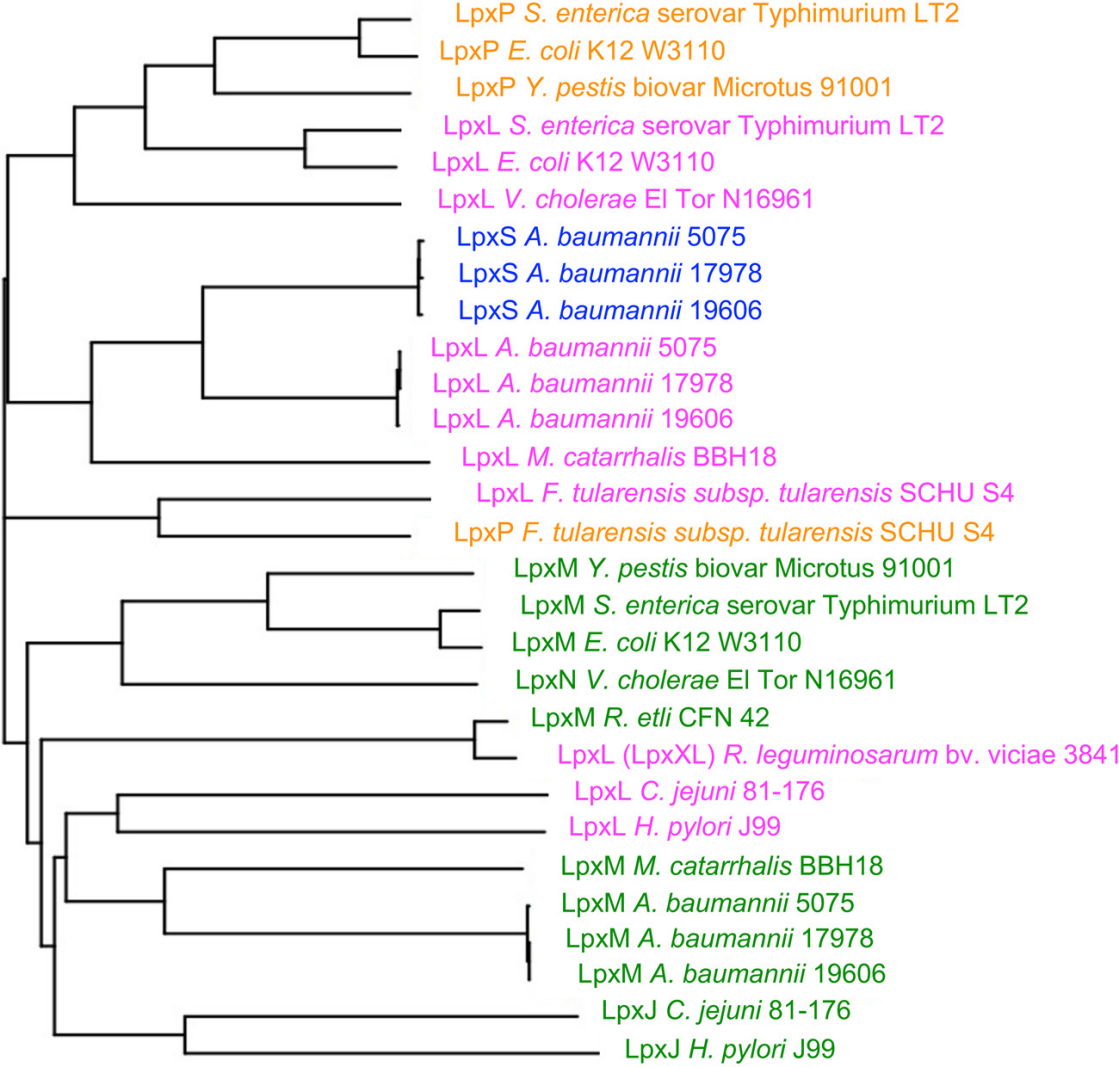
Distribution of LpxS among known secondary lipid A acyltransferases. Selected protein sequences from the BioCyc database were aligned using Clustal Omega, and the multiple sequence alignment output is shown as a dendrogram. Different lipid A acyltransferase homologues are represented by different colors. Blue, orange, and magenta represent acylation at the 2′ position by LpxS, LpxP, and LpxL, respectively. Green indicates acylation at the 3′ position by LpxM and other closely related acyltransferases (LpxJ and LpxN).

This genomic variability can be an outcome of horizontal gene transfers via mobile elements and genomic rearrangements, such as gene duplication and amplification (27–29). Often, acquired genes provide a selective benefit toward bacterial adaptation under diverse growth conditions (30). We determined how closely related A. baumannii LpxS is to known late acyltransferases in other Gram-negative bacteria (Fig. 2). A. baumannii LpxS and LpxL are clustered in the same clade and share more similarity with LpxL from Moraxella catarrhalis. A similar case is observed for LpxM of both organisms, although they are grouped in a separated clade. The distribution is understandable given that Acinetobacter and *Moraxella* belong to the same family (18). LpxLs from other Gram-negative bacteria are more distantly related and grouped in different clades. LpxL and LpxP represent an event of gene duplication where both encode lipid A acyltransferases but differ in substrate donor specificity and are differentially regulated (7, 31, 32). Overall, with the exception of LpxXL from Rhizobium leguminosarum and LpxJ (LpxL-like enzyme) from Helicobacter pylori and Campylobacter jejuni, we observed LpxS and respective additional 2′ secondary acyltransferases (LpxL, LpxP) clustered separately from LpxM acyltransferases. We concluded LpxS is likely a result of an LpxL gene duplication based on these observations.

### LpxS is functionally comparable to LpxL when expressed in E. coli.

E. coli encodes four lipid A secondary acyltransferases. LpxL, LpxM, and LpxP (Fig. 1A) all function at the cytoplasmic surface of the inner membrane as part of the conserved lipid A biosynthetic pathway and utilize acyl-acyl carrier proteins as the fatty acid donor (4, 7), whereas PagP is an outer membrane enzyme that incorporates a palmitate (C16:0) at the 2 position and is activated in response to outer membrane stress. Unlike LpxL/M/P, PagP is considered a lipid A modification enzyme and uses a glycerophospholipid as the donor substrate (33). Given the well-characterized biosynthetic pathways of E. coli, heterologous expression of putative lipid A synthesis or modification genes in this organism has been a useful tool for functional characterization of gene products. Thus, the *A1S_1255* (*lpxS*) open reading frame (ORF) was cloned into expression vector pMMB67EH, and the resulting plasmid, pLpxS, was expressed in the W3110 Δ*lpxL* (Δ*lpxL*_Ec_) or Δ*lpxM* (Δ*lpxM*_Ec_) backgrounds.

**FIG 3:**
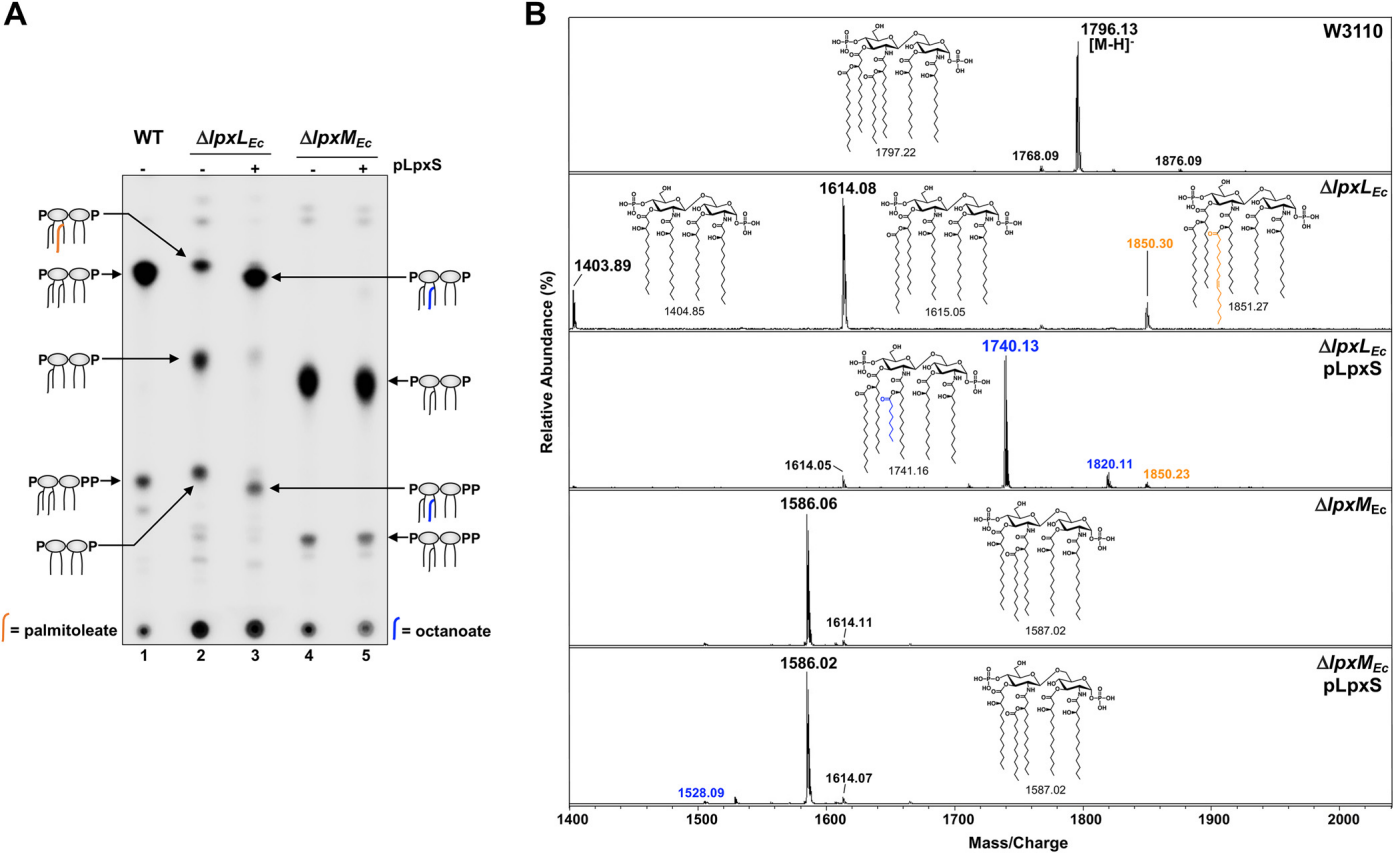
LpxS exhibits lipid A late acyltransferase activity in E. coli. (A) TLC analysis of lipid A from E. coli strains expressing LpxS. Wild-type W3110, Δ*lpxL*_Ec_, and Δ*lpxM*_Ec_ strains with or without pLpxS were grown to mid-log in minimal medium containing ^32^P_i_ at 30°C. Radiolabeled lipid A species (depicted as cartoons) were isolated, separated by TLC, and visualized by phosphorimaging. LpxS expression promoted lipid A acylation at the 2′ position. (B) MALDI-TOF mass spectrometry of lipid A from E. coli expressing LpxS. Lipid A samples were analyzed in the reflectron negative mode and indicated LpxS-dependent octanoate addition. Lipid A chemical structures and corresponding exact masses are provided for reference. Lipid A harboring an octanoate (C8:0) or palmitoleate (C16:1) acyl group at the 2′ position is represented in blue or orange, respectively. The peak values are the mass-to-charge ratio of each lipid A species detected, and spectra are representative of three independent biological repetitions. Data in both panels are representative of three biological repetitions.

Previous studies determined that Δ*lpxL*_Ec_ exhibits an altered growth phenotype exhibiting temperature sensitivity in nutrient broth. Growth on minimal medium at lower temperatures slows cellular metabolism, permitting translocation of LPS with reduced acylation to the OM (34). Therefore, cells were grown at 30°C in M9 minimal medium supplemented with ^32^P_i_, and the lipid A species was analyzed by thin-layer chromatography (TLC) afterward. As reported earlier, the Δ*lpxL*_Ec_ strain synthesized a mixture of hexa-, penta-, and tetra-acylated lipid A forms (Fig. 3A, lane 2) (35). Since LpxL function is required for efficient LpxM acyltransferase activity in E. coli, tetra-acylated lipid A (Fig. 1) can be easily detected by TLC (35). Partial LpxM activity results in addition of a myristoyl group (C14:0) to form the penta-acylated species. The relative abundance of hexa-acylated lipid A in the Δ*lpxL*_Ec_ strain can be explained as a compensatory function of LpxP followed by LpxM acylation (34).

Upon expression of LpxS_Ab_, the majority of penta-acylated lipid A disappears and hexa-acylation becomes predominant. This suggests that LpxS-dependent acylation occurs at the 2′ position replacing LpxL and restores full LpxM activity in the cell (Fig. 3A, lane 3). However, when pLpxS was expressed in the Δ*lpxM*_Ec_ strain, migration of penta-acylated forms was unchanged (Fig. 3A, lanes 4 and 5), supporting the notion that LpxS functionally replaces E. coli LpxL. We also evaluated LpxS activity in a strain background lacking all four E. coli secondary acyltransferases (W3110 Δ*lpxL* Δ*lpxM* Δ*lpxP* Δ*pagP* tetra-mutant: W3Δ4X) and confirmed that LpxS transferred a single secondary acyl chain (Fig. S2A). Therefore, LpxS is specific to the 2′ position, similar to LpxL, and is monofunctional.

10.1128/mBio.01295-21.2 FIG S2 LpxS attaches a single secondary acyl chain. (A) TLC analysis of ^32^P-labeled lipid A from the E. coli strains W3Δ*lpxL*, Δ*lpxM*, Δ*lpxP*, and Δ*pagP* (W3Δ4X) tetra-acylated mutant expressing LpxS. Cells were grown in the presence of ^32^P_i_ at 30°C followed by lipid A extraction. Lipid A species are depicted as cartoons, with the octanoate fatty acid colored blue. (B) MALDI-TOF mass spectrometry of lipid A from W3Δ4X with or without LpxS expression. Lipid A samples were analyzed in the reflectron negative mode and indicate LpxS-dependent octanoate addition. Octanoylated lipid A species are indicated in blue, and chemical structures and exact masses are shown. Download FIG S2, PDF file, 0.3 MB. Copyright © 2021 Herrera et al. 2021 Herrera et al. https://creativecommons.org/licenses/by/4.0/ This content is distributed under the terms of the Creative Commons Attribution 4.0 International license.

### LpxS is an octanoyl (C8:0) acyltransferase.

To determine the length of the acyl group transferred by LpxS, we performed matrix-assisted laser desorption ionization–time of flight (MALDI-TOF) mass spectrometry. Lipid A was purified from E. coli grown under the same conditions as in Fig. 3A but in the absence of ^32^P_i_. Wild-type W3110 lipid A showed peaks at *m/z* 1796.13 and 1876.09 corresponding to hexa-acylated *bis*-phosphorylated lipid A and a species containing a diphosphate at the 1 position, respectively (Fig. 3B). The 1-diphosphate lipid A arose from the action of LpxT, an inner membrane kinase that transfers a phosphate from undecaprenyl pyrophosphate to lipid A (36, 37). LpxT-modified species are also visible by TLC (Fig. 3A). As expected from the TLC profile, the Δ*lpxL*_Ec_ strain synthesized tetra-acylated (*m/z* 1403.89), penta-acylated (*m/z* 1614.08), and hexa-acylated (*m/z* 1850.30) lipid A variants (Fig. 3B). The minor molecular ion at *m/z* 1850.30 resulted from the addition of a palmitoleoyl (C16:1) acyl chain by LpxP followed by acylation by LpxM, confirming the compensatory LpxP activation at 30°C. LpxS expression in the Δ*lpxL*_Ec_ strain yielded a predominant hexa-acylated species at *m/z* 1740.13 (Fig. 3B). Surprisingly, the difference due to the acyltransferase activity is 126 arbitrary mass units (amu) corresponding to an octanoate acyl chain (C8:0). Further, a minor peak at *m/z* 1820.11 represents the 1-diphosphate hexa-acylated derivative containing the C8:0 chain. Both Δ*lpxM*_Ec_ strain spectra, with or without LpxS expression, confirmed production of penta-acylated lipid A lacking a myristate (C14:0) at the 3′ position (*m/z* ∼1586) (Fig. 3B). Very minor octanoylated lipid A at *m/z* 1528.09 was detected in the Δ*lpxM*_Ec_ strain upon expression of LpxS. Expression of LpxS in W3Δ4X, a strain lacking all secondary acyltransferases, resulted in a major peak at *m/z* 1529.13 corresponding to a single octanoic acyl chain added to tetra-acylated *bis*-phosphorylated lipid A (Fig. S2B). In summary, our TLC and mass spectrometry analysis in E. coli determined that *A1S_1255* encodes a functional secondary acyltransferase that specifically adds a single octanoate to the primary linked fatty acid at the 2′ position. To our knowledge, A1S_1255 transfers the shortest secondary acyl chain reported, and for that reason, we selected the enzyme name “LpxS,” where “S” indicates short.

### LpxS is a functional acyltransferase in A. baumannii.

In A. baumannii, both LpxL and LpxM transfer C12:0 chains to lipid A. LpxL is a monofunctional enzyme, while LpxM is bifunctional, generating a mixture of hexa- and hepta-acylated LPS forms in the OM (Fig. 1B) (23). These species can be modified by the enzyme LpxO, an oxygenase that adds a hydroxyl group to the LpxL-derived secondary acyl chain at the 2′ position, resulting in 4 major lipid A species in this organism. Notably, under standard laboratory conditions (rich medium, 37°C) A. baumannii lipid A does not contain a C8:0 fatty acid (23, 38). Here, we investigated whether ectopic expression of LpxS could alter A. baumannii lipid A structure during growth in LB at 37°C by both TLC and mass spectrometry.

**FIG 4:**
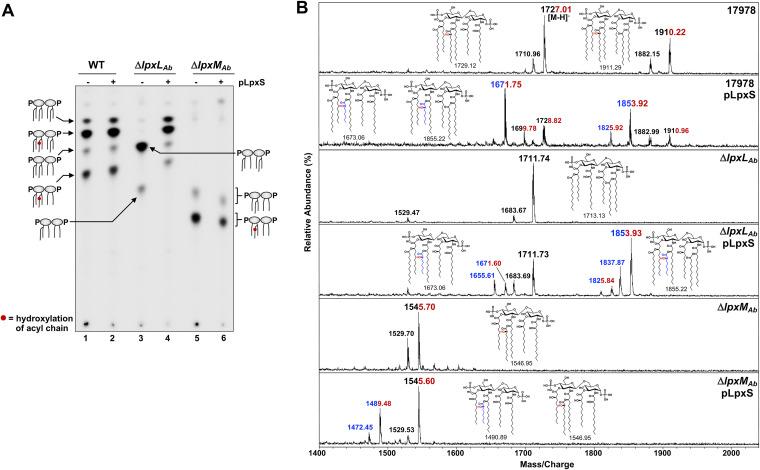
Expression of LpxS in A. baumannii promotes lipid A octanoylation during growth at 37°C. (A) TLC analysis of radiolabeled lipid A from A. baumannii strains expressing LpxS. Wild-type 17978, Δ*lpxL*_Ab_, and Δ*lpxM*_Ab_ strains with or without pLpxS were grown to mid-log in LB containing ^32^P_i_ at 37°C. Lipid A species are depicted as cartoons. (B) MALDI-TOF mass spectrometry of lipid A from A. baumannii expressing LpxS in the reflectron negative mode. Chemical structures and corresponding exact masses are provided for reference. Spectra are representative of three independent biological repetitions. Numbers in blue represent octanoylated (C8:0) species, and red indicates hydroxylation of the 2′-secondary acyl chain by LpxO. Data in both panels are representative of three biological repetitions.

Lipid A profiles were evaluated in 17978 wild-type (WT), Δ*lpxL* (Δ*lpxL*_Ab_), and Δ*lpxM* (Δ*lpxM*_Ab_) strains carrying plasmid pLpxS. LpxS overexpression did not modify the TLC lipid A pattern observed in WT showing the typical 4 lipid A species (Fig. 4A, lanes 1 and 2). We considered three possible explanations. First, LpxS is not functional under the evaluated growth conditions. Second, LpxS is functional but other late acyltransferase activities are dominant. Third, a difference between lipid A species containing either a C8:0 or a C12:0 could not be clearly resolved by TLC. Unlike the Δ*lpxL*_Ec_ strain, the Δ*lpxL*_Ab_ strain is capable of growing at temperatures above 32°C, and no compensatory late acyltransferase activity (e.g., LpxP) was observed (Fig. 4A, lane 3) (23). Expression of LpxS in the Δ*lpxL*_Ab_ strain resulted in both hexa- and hepta-acylated lipid A species (Fig. 4A, lane 4), indicating LpxS activity. When expressed in the Δ*lpxM*_Ab_ strain, a strain that produces only penta-acylated lipid A, we could detect a slight shift in TLC migration but the same number of acyl chains (Fig. 4A, lanes 5 and 6). In all strains expressing LpxS, LpxO hydroxylation did not appear to be disrupted. Overall, these data support the idea that LpxS acylates A. baumannii lipid A exclusively at the 2′ position.

We further characterized the observed lipid A species by mass spectrometry. Strain 17978 showed two main peaks at *m/z* 1910.22 and 1727.01, corresponding to hydroxylated hepta- and hexa-acylated lipid A forms, respectively (Fig. 4B). Ions representing nonhydroxylated forms were also present, as well as species differing by 28 amu arising from a two-carbon difference in a single fatty acyl chain. Importantly, LpxS overexpression produced additional major peaks at *m/z* 1853.92 and 1671.75 representing the presence of 2′-hydroxyoctanoate in hepta- and hexa-acylated lipid A, respectively. As previously reported (23), the Δ*lpxL*_Ab_ strain synthesized a major peak at *m/z* 1711.74 consistent with the loss of a secondary lauroyl group at the 2′ position (Fig. 4B). LpxS expressed in the Δ*lpxL*_Ab_ strain fully restored acylation with both hepta-acylated (e.g., *m/z* 1853.93) and hexa-acylated (e.g., *m/z* 1671.60) species harboring octanoic fatty chains. These data confirm that LpxS acylates lipid A at the 2′ position in A. baumannii and correspond well with the observed TLC profiles (Fig. 4A). The Δ*lpxM*_Ab_ strain lipid A generated the predominant ions at *m/z* 1545.70 and 1529.70 corresponding to hydroxylated and nonhydroxylated penta-acylated species, respectively. A new hydroxylated penta-acylated species at *m/z* 1489.48 was observed upon LpxS expression that corresponded to a 2′ octanoate (Fig. 4B). Taken together, these results confirm that LpxS transfers an octanoic fatty acyl chain to the 2′-linked primary acyl chain of A. baumannii LOS. Further, LpxO-dependent hydroxylation at the 2′ position was not impacted by the length of fatty acyl chain.

### Endogenous *lpxS* expression is induced during growth at low temperature.

**FIG 5:**
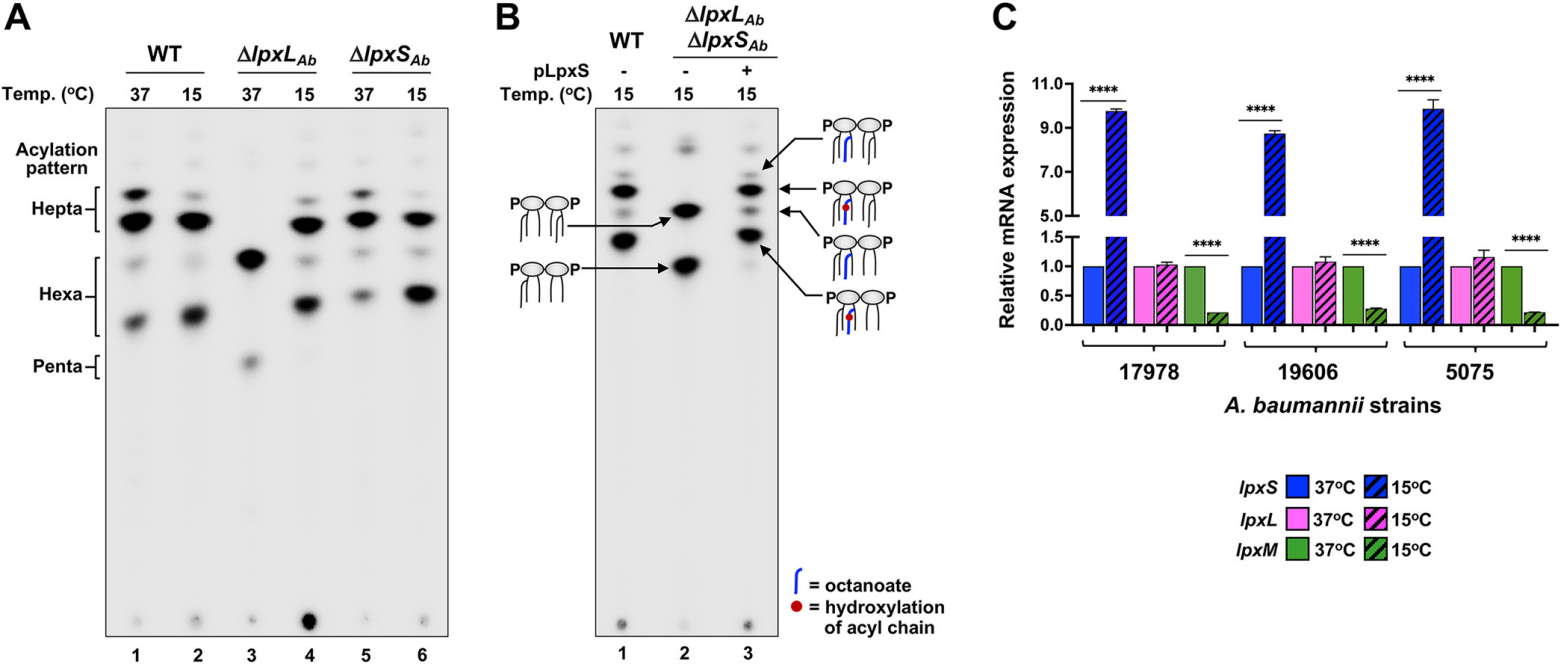
Native *lpxS* expression is induced under cold conditions. (A and B) TLC analysis of radiolabeled lipid A from A. baumannii strains grown in LB at either 37°C or 15°C. Lipid A species are depicted as cartoons. (C) Transcription levels of genes that encode late acyltransferases were evaluated by qRT-PCR from A. baumannii (strains 17978, 19606, and 5075) grown in LB medium at 37°C and 15°C that was harvested at log phase. Levels of *lpxS* (blue), *lpxL* (magenta), and *lpxM* (green) transcripts at 15°C are relative to 37°C and normalized using *gyrA* as the reference gene. Statistical significance (****) was calculated using *t* test (*P* < 0.001). Data represent the average from three biological repetitions.

Given that ectopically expressed LpxS is functional and adds a much shorter fatty acyl chain, we considered the possibility that LpxS may function like E. coli LpxP during cold shock (7). To assess whether cold temperature promoted endogenous LpxS activity, ^32^P-labeled wild-type, Δ*lpxL*_Ab_, and Δ*lpxS*_Ab_ cells were grown at 37°C or 15°C followed by lipid A analysis. Overall, WT cells displayed similar lipid A migration patterns at the two temperatures (Fig. 5A, lanes 1 and 2), but with a notable increase in hexa-acylated forms at low temperature. Interestingly, both hexa- and hepta-acylated lipid A variants were recovered in the Δ*lpxL*_Ab_ mutant when grown at 15°C (lane 4) compared to the 37°C lipid A profile. The Δ*lpxS*_Ab_ mutant, on the other hand, synthesized similar lipid A variants at the two temperatures (lanes 5 and 6). Taken together, one interpretation of these results is that activation of chromosomal *lpxS* at 15°C restores loss of the secondary acyl chain in the Δ*lpxL*_Ab_ strain, and both LpxL and LpxS are typically expressed at low temperatures. An alternative explanation, although unlikely, is that LpxM activity is increased at low temperature and adds 3 acyl chains to lipid A. To elucidate whether lipid A acyltransferases exhibit mutual compensatory effects under cold conditions, an Δ*lpxL*_Ab_ Δ*lpxS*_Ab_ double mutant was evaluated. In this background, there is a complete loss of hepta-acylated species at both growth temperatures. LpxS overexpression in the double mutant restored WT acylation patterns (Fig. 5B and Fig. S3). Thus, we concluded that LpxS and LpxL are both active at 15°C and LpxM does not exhibit a compensatory acylation.

10.1128/mBio.01295-21.3 FIG S3 Lipid A profile of the Δ*lpxL*_Ab_ Δ*lpxS*_Ab_ double mutant grown in LB medium at 37°C. The double mutant exhibits a similar lipid A phenotype at 37°C (lane 2) and at 15°C (Fig. 5B, lane 2). LpxS overexpression restores lipid A acylation similar to WT (lanes 1 and 3). Lipid A samples were separated by TLC. Lipid A species are depicted as cartoons with octanoate in blue and hydroxylation by LpxO in red. Download FIG S3, PDF file, 0.3 MB. Copyright © 2021 Herrera et al. 2021 Herrera et al. https://creativecommons.org/licenses/by/4.0/ This content is distributed under the terms of the Creative Commons Attribution 4.0 International license.

**FIG 6:**
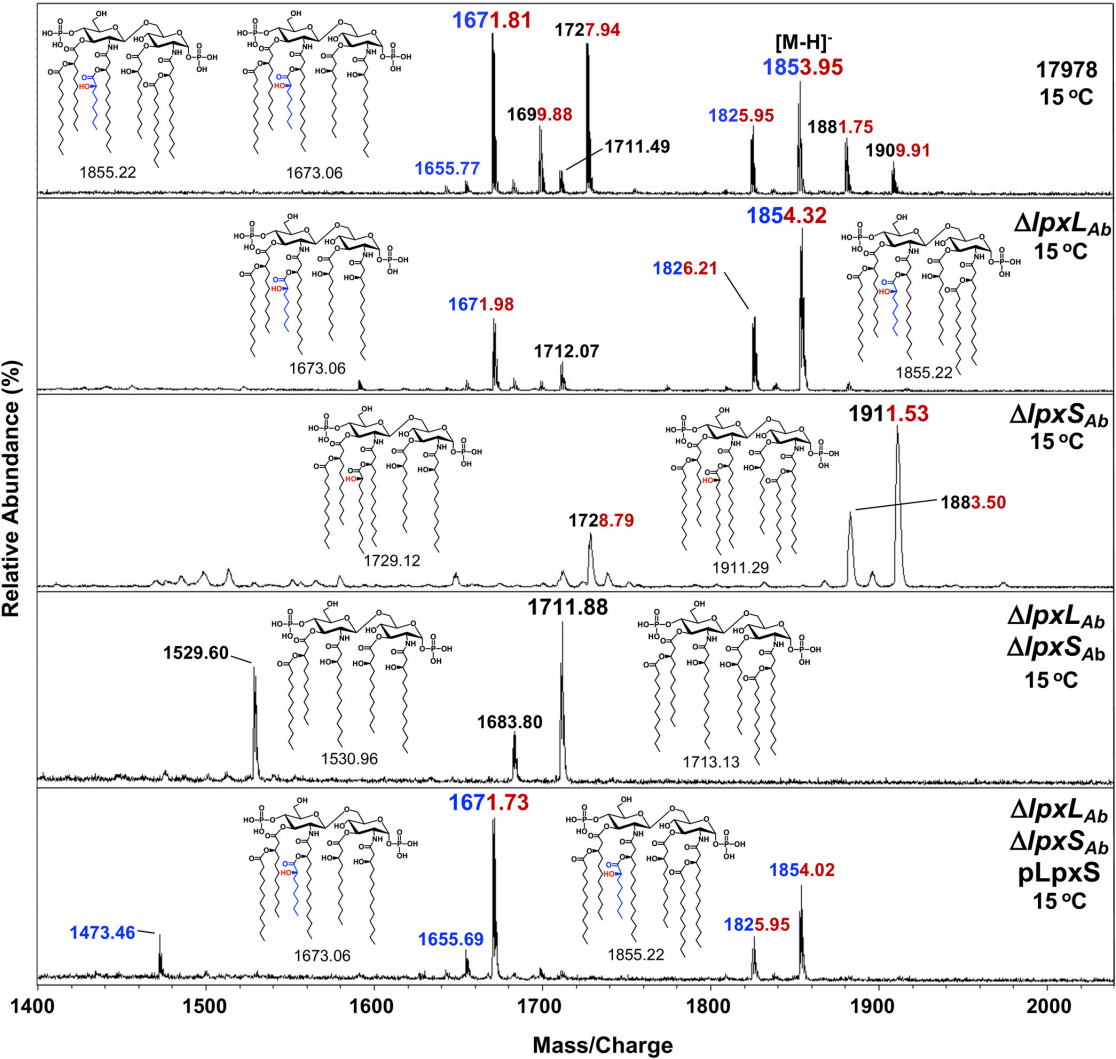
MALDI-TOF mass spectrometry of lipid A from A. baumannii acyltransferase mutants under cold conditions. Cells were grown in LB medium at 15°C. Addition of an octanoyl group is evident in strains expressing LpxS. Lipid A chemical structures and their corresponding exact mass are indicated. Peak values in blue indicate lipid A species harboring an octanoate (C8:0). Red indicates hydroxylation by LpxO. Data are representative of three biological repetitions.

Next, we determined the lipid A species produced under low-temperature growth by mass spectrometry. The WT showed abundant hydroxylated hexa- and hepta-acylated lipid A species harboring an octanoic acyl chain at *m/z* 1671.81 and 1853.95, respectively (Fig. 6). Lipid A containing C12:0 added by LpxL was also present (e.g., *m/z* 1727.94 and 1909.91). These results confirmed LpxS and LpxL are active at 15°C, and cells synthesize a mixture of lipid A forms acylated with C8:0 and C12:0 at the 2′ position (Fig. 6). This is similar to what is seen in E. coli during cold shock, with a mixture of C12:0 and C16:1 addition by LpxL and LpxP, respectively (7). In the *lpxL* mutant, growth at 15°C resulted in essentially 100% C8:0 addition with major peaks for hydroxyl-hepta-acylated species (*m/z* 1854.32 and 1826.21) and hydroxyl-hexa-acylated species (*m/z* 1671.98). This suggests that LpxS activity is not limited by the acyl-donor pool but rather competes with LpxL during lipid A biosynthesis (Fig. 6 and Fig. S4). All octanoyl groups were absent in either the Δ*lpxS*_Ab_ or Δ*lpxL*_Ab_ Δ*lpxS*_Ab_ strain at 15°C but could be restored upon plasmid complementation. Growth at 37°C did not result in C8:0 addition, unless LpxS was ectopically expressed (Fig. 4 and Fig. S4). On the whole, our analysis confirmed that LpxS is activated at low temperature, competing with LpxL. Further, low temperature did not alter selectivity for donor substrate and specificity for target substrate in the A. baumannii acyltransferases.

10.1128/mBio.01295-21.4 FIG S4 MALDI-TOF mass spectrometry spectra from Δ*lpxS*_Ab_ complemented strain and Δ*lpxL*_Ab_ Δ*lpxS*_Ab_ double mutant overexpressing LpxS were evaluated and compared to their respective control strains. Numbers in blue show octanoylated lipid A whereas red indicates hydroxylated acyl chain at the 2′ position. Download FIG S4, PDF file, 0.6 MB. Copyright © 2021 Herrera et al. 2021 Herrera et al. https://creativecommons.org/licenses/by/4.0/ This content is distributed under the terms of the Creative Commons Attribution 4.0 International license.

Given LpxS conservation within A. baumannii species and the observed temperature-dependent changes in lipid A acylation, we evaluated whether genes encoding the A. baumannii acyltransferases are temperature regulated. Therefore, *lpxS*, *lpxL*, and *lpxM* transcription was determined at different temperatures in 17978 and two additional commonly used laboratory strains, 19606 and 5075. Real-time quantitative reverse transcription-PCR (qRT-PCR) analysis showed *lpxS*_Ab_ transcripts were highly upregulated at 15°C compared to 37°C. *lpxS*_Ab_ transcripts increased 9.7-fold in 17978, 8.7-fold in 19606, and 9.8-fold in 5075 (Fig. 5C). Surprisingly, *lpxL*_Ab_ transcript levels were similar at the two temperatures for all strains (Fig. 5C). Expression of *lpxM*_Ab_ significantly decreased 4.8-fold, 3.6-fold, and 4.5-fold in 17978, 19606, and 5075, respectively (Fig. 5C). These results are consistent with lipid A profiles. LpxS_Ab_ synthesis was increased at low temperatures to efficiently compete with LpxL_Ab_ activity for target substrate. The fact that *lpxM*_Ab_ is downregulated at 15°C correlates with a visible increase in hexa-acylated glycoforms in comparison to lipid A species from cells grown at 37°C. Importantly, the decreased expression of *lpxM*_Ab_ was in response to cold temperature and independent of either *lpxS* or *lpxL* synthesis (Fig. S5A and B).

10.1128/mBio.01295-21.5 FIG S5 Controls for RT-qPCR analysis of acyltransferase gene expression. (A) Controls for mutant and complemented strains. *lpxS* transcript levels determined in the Δ*lpxS*_Ab_ strain and the Δ*lpxL*_Ab_ Δ*lpxS*_Ab_ double mutant carrying empty plasmid or pLpxS relative to WT 17978. Cells were grown in LB medium at 15°C. (B) *lpxM* expression is downregulated in response to cold temperature independently of other acyltransferase gene deletions. Δ*lpxS*_Ab_ and Δ*lpxL*_Ab_ Δ*lpxS*_Ab_ mutants carrying empty plasmid or overexpressing LpxS were grown in LB at 37°C and 15°C. Ratio of *lpxM* transcript levels at 15°C is relative to that at 37°C. Levels of transcripts are normalized using *gyrA* as the reference gene. Data represent the average from three biological repetitions. Statistical significance (****) was calculated using *t* test (*P < *0.001). Download FIG S5, PDF file, 0.4 MB. Copyright © 2021 Herrera et al. 2021 Herrera et al. https://creativecommons.org/licenses/by/4.0/ This content is distributed under the terms of the Creative Commons Attribution 4.0 International license.

### Lipid A octanoylation supports the outer membrane permeability barrier during low-temperature growth.

**FIG 7:**
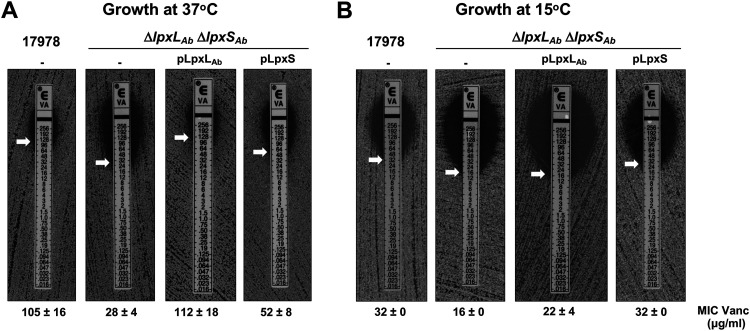
LpxS-dependent acylation contributes to the outer membrane permeability barrier. Vancomycin resistance was used as a proxy of outer membrane barrier function. The MIC of vancomycin was determined for WT 17978 and the Δ*lpxL*_Ab_ Δ*lpxS*_Ab_ double mutant carrying empty plasmid (p) or plasmids individually expressing either LpxS or LpxL_Ab_. The MIC is indicated by a white arrow. Cells were grown at 37°C (A) and 15°C (B) on LB agar plates. Data in both panels are representative of three biological repetitions.

The amphipathic nature of LPS/LOS contributes to the permeability barrier of the OM (2), providing resistance to toxic molecules, including the large hydrophilic antibiotic vancomycin. Since increased sensitivity to vancomycin indicates changes in OM permeability (2, 39), we determined how different lipid A structures might influence resistance at different growth temperatures using the Δ*lpxL*_Ab_ Δ*lpxS*_Ab_ double mutant ectopically expressing either LpxL_Ab_ or LpxS. E-strips were used to determine the MIC of cells grown at 37°C and 15°C. The WT was highly resistant to vancomycin at 37°C, displaying a MIC of 105 ± 16 μg/ml, whereas the Δ*lpxL*_Ab_ Δ*lpxS*_Ab_ strain showed a MIC reduction of 3.75-fold (Fig. 7A). LpxL_Ab_ expression restored resistance to WT levels (112 ± 18 μg/ml) at 37°C, while LpxS_Ab_ synthesis only partially recovered vancomycin resistance (52 ± 8 μg/ml). Cells grown at 15°C showed the opposite pattern of resistance. The vancomycin potency increased over 3-fold in strains grown under cold conditions, a finding previously reported by the Brown laboratory for E. coli K-12 (Fig. 7) (40). Etest showed that LpxL_Ab_ synthesis only partially restored resistance of the double mutant, but LpxS_Ab_ expression fully restored the MIC to WT levels (Fig. 7B). We also tested antibiotic resistance using a second large-scaffold antibiotic, novobiocin, and those data reinforced our findings with vancomycin (Table S2). Overall, the presence of a shorter acyl chain during growth under cold conditions increased the effectiveness of the OM permeability barrier.

10.1128/mBio.01295-21.8 TABLE S2 Novobiocin MIC. Download Table S2, PDF file, 0.03 MB. Copyright © 2021 Herrera et al. 2021 Herrera et al. https://creativecommons.org/licenses/by/4.0/ This content is distributed under the terms of the Creative Commons Attribution 4.0 International license.

### Shorter acyl-chain addition by LpxS reduces TLR4-dependent immune response.

The lipid A anchor of LPS or LOS is recognized by the TLR4/MD2 complex of the innate immune system, leading to a robust signaling cascade and cytokine production. Variation in the lipid A chemical structure, such as the removal or modification of phosphate groups as well as the number, length, or position of esterified acyl chains, can produce differential inflammatory responses to LPS/LOS (24, 41, 42). In fact, modified lipid A forms are already in use as vaccine adjuvants. Previously, our group developed a system for combinatorial structural diversification of E. coli lipid A, producing a wide range of lipid A molecules with adjuvant potential (42). As LpxS transfers a very short acyl chain, we questioned whether replacement of a C12:0 fatty acid with a C8:0 residue could alter TLR4/MD2 activation, aiding in adjuvant design.

**FIG 8:**
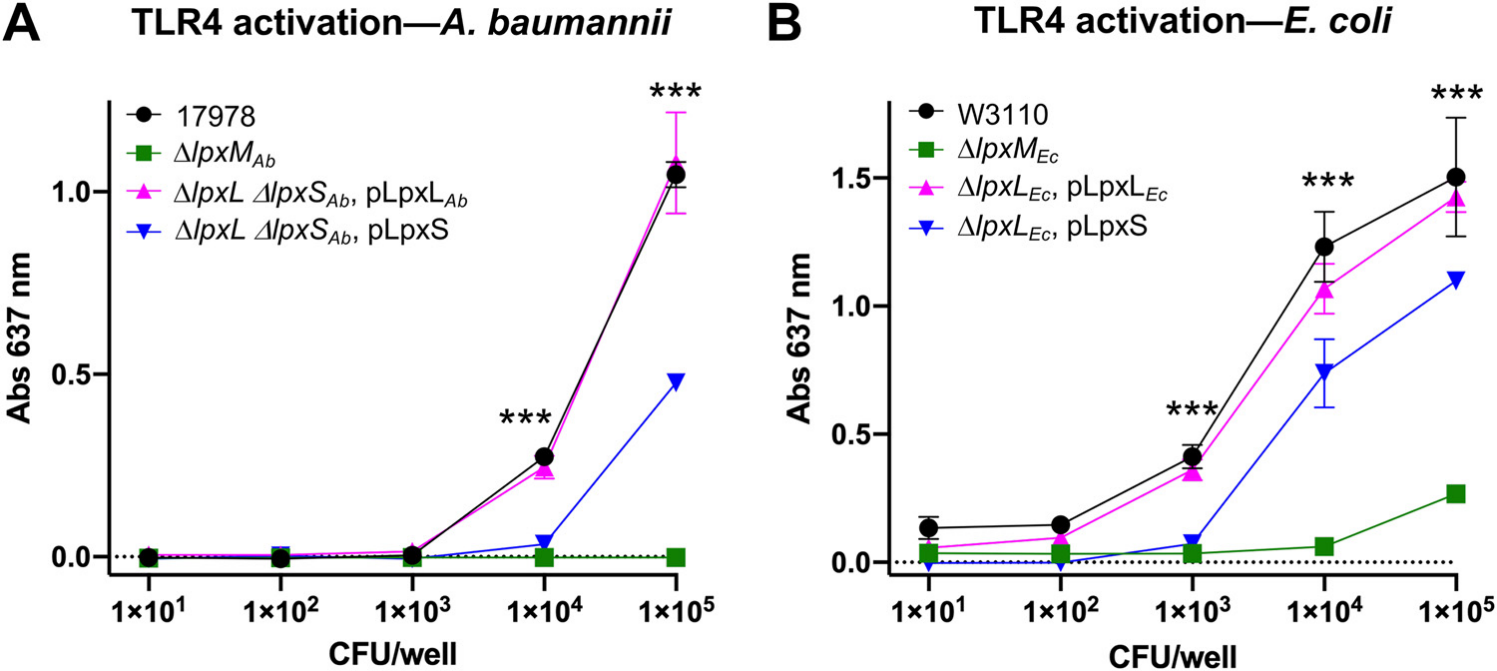
Octanoate instead of laurate at the 2′ position induces a differential TLR4 activation. (A) HEK-Blue hTLR4 cells were challenged with lysates from CFU serial dilutions of WT 17978, the Δ*lpxM*_Ab_ mutant, and the Δ*lpxL*_Ab_ Δ*lpxS*_Ab_ double mutant carrying either pLpxL_Ab_ or pLpxS. (B) Lysates from the E. coli strains WT W3110, Δ*lpxM*_Ab_, and Δ*lpxL*_Ab_ harboring plasmid pLpxL_Ec_ or pLpxS were used. TLR4 induction was measured using Quanti-Blue. Data in both panels represent the average from three biological repetitions. Statistical significance (***) was determined by two-way ANOVA and calculated using GraphPad Prism version 9 (*P* < 0.001).

Whole-cell preparations from both A. baumannii and E. coli were screened using HEK-Blue hTLR4 cells. In this cell line, LPS/LOS binding to the TLR4-MD2-CD14 complex activates NF-κB and the activator protein 1, which coordinately induces production of the secreted embryonic alkaline phosphatase (SEAP) reporter. HEK-Blue hTLR4 cells were treated with a range of CFU dilutions of each bacterial sample. A. baumannii lipid A species in the Δ*lpxL*_Ab_ Δ*lpxS*_Ab_ double mutant expressing LpxL_Ab_ stimulated TLR4 almost identical to that of WT 17978 (Fig. 8A). Octanoate instead of laurate at the 2′ position of lipid A showed a significant reduction in TLR4 response (Fig. 8A). Lipid A from a negative-control strain lacking *lpxM*_Ab_ was markedly less effective, as previously reported (Fig. 8A) (23). A similar trend was seen when using engineered E. coli strains. Lipid A from WT and complemented Δ*lpxL*_Ec_ strains both showed a robust activation of TLR4 as hexa-acylated, *bis*-phosphorylated E. coli lipid A induces a strong proinflammatory response (42) (Fig. 8B). Replacement of the LpxL C12:0 chain with a C8:0 (Δ*lpxL*_Ec_, pLpxS) resulted in a significantly lower TLR4 response relative to the WT E. coli lipid A structure but higher than that of the penta-acylated Δ*lpxM*_Ec_ mutant (Fig. 8B). Control assays using HEK-Blue Null2 cells lacking hTLR4 confirmed that the SEAP induction in Fig. 8 was specific to TLR4 activation (Fig. S6). Collectively, these data show that acyl chain length at the 2′ position is relevant for induction of the TLR4 pathway and that LpxS could prove useful for LPS adjuvant engineering.

10.1128/mBio.01295-21.6 FIG S6 Controls for TLR4 assays. Cells from strains (A) WT 17978, Δ*lpxM*_Ab_, and Δ*lpxL*_Ab_ Δ*lpxS*_Ab_ overexpressing LpxS_Ab_ or LpxL_Ab_ and (B) WT W3110, Δ*lpxM*_Ec_, Δ*lpxL*_Ec_ pLpxL_Ec_, and Δ*lpxL*_Ec_ pLpxS showed no induction on null HEK293 cells that served as negative control for TLR4. (C) Purified LPS from E. coli (agonist) and R. sphaeroides (antagonist) was evaluated at different concentrations for TLR4 stimulation in the reporter HEK-Blue hTLR4 cells. Download FIG S6, PDF file, 0.2 MB. Copyright © 2021 Herrera et al. 2021 Herrera et al. https://creativecommons.org/licenses/by/4.0/ This content is distributed under the terms of the Creative Commons Attribution 4.0 International license.

## DISCUSSION

To maintain optimal fluidity and permeability properties of the membrane, cells must alter lipid composition in a process known as homeoviscous adaptation. Bacteria have evolved multiple strategies to combat membrane rigidification including the introduction of branched-chain fatty acids, altering the length of fatty acyl chains, and, most commonly, increasing the level of lipids with unsaturated fatty acids (9, 10). However, in comparison to changes in glycerophospholipid content, little is known about structural alterations of LPS under cold conditions. The best example is the cold induction of the lipid A acyltransferase LpxP that replaces a C12:0 fatty acid with a C16:1, increasing outer membrane fluidity (7, 8). Here, we demonstrate that A. baumannii takes a different approach, and instead of incorporating an unsaturated fatty acid, the bacterium exchanges a C12:0 fatty acid (Fig. 5 and 6) with a short, C8:0 chain. Reduction of the acyl chain length would decrease the Van der Waals molecular interactions between neighboring LPS molecules, resulting in a more fluid membrane (2, 43). Although associated primarily with hospital-acquired infections (44), A. baumannii can be found in a variety of reservoirs (45, 46) with environmental isolates capable of surviving in a wide range of temperatures for prolonged periods of time, including in the cold (47). Thus, the bacterium must be able to maintain membrane fluidity under a variety of conditions.

Previously, our group characterized the LpxL and LpxM lipid A acyltransferases of A. baumannii. Here, using both genetic and biochemical approaches, we describe LpxS, a cold-induced LpxL homologue that transfers the shortest known secondary acyl chain to lipid A. LpxL has shown multiple events of gene duplications during evolution of Gram-negative bacteria (48). A. baumannii LpxS and LpxL acyltransferases are clustered in the same clade, indicating the closest relationship. Similar grouping between LpxP and LpxL is observed in other Gram-negative bacteria considered in our analysis. Other lipid A acyl transferases such as LpxM, LpxJ, and LpxN are more distantly related and grouped in separated clades (Fig. 2). These enzymes have maintained their lipid A acyltransferase function but diverged in their selectivity for acyl donor substrates (49, 50), the site specificity of acylation, and whether Kdo glycosylation is required for enzymatic activity (5, 51).

Secondary acyltransferases (with the exception of PagP) (33) primarily utilize acyl-acyl carrier proteins (ACPs) as donor substrates that are generated by the fatty acid synthase (FASII) pathway ranging from 10 to 16 carbons in length (4, 24, 52, 53). A unique case is the incorporation of a very long acyl chain (27-hydroxyoctacosanoic acid) to lipid A in *Rhizobium* (24, 54) that notably requires a dedicated acyl carrier protein termed ACP-XL. Whether LpxS uses a dedicated ACP donor expressed under cold conditions remains unclear. Nevertheless, this seems doubtful, given that LpxS functions efficiently in E. coli at 30°C, and suggests that the enzyme uses octanoyl-ACP from the general acyl-ACP pool. Octanoyl-ACP should be readily available as it is required for synthesis of lipoic acid, an important enzyme cofactor that is found in all domains of life (52).

LpxS easily restored complete lipid A synthesis in E. coli lacking *lpxL* at 30°C, yet octanoylation was barely detectable when LpxS was ectopically expressed in Δ*lpxM*_Ec_ (Fig. 3). Contrary to this, in A. baumannii, plasmid expression of LpxS led to efficient incorporation of C8:0 even when LpxL_Ab_ was present (Fig. 4 and Fig. S4). What accounts for these differences? Possibly, LpxS is less stable in E. coli during growth at 30°C or less octanoyl-ACP is available as a substrate donor. Still, multiple lines of evidence using heterologous expression in multiple E. coli and A. baumannii mutants clearly demonstrate that LpxS transfers a single acyl chain to lipid A and is induced at cold temperatures.

LpxS expression increased by ∼10-fold across multiple strain backgrounds during growth under cold conditions. Interestingly, *lpxL* transcripts did not change, but *lpxM* transcripts decreased ∼4-fold. Indeed, we also noticed a reproducible decrease in the amount of hepta-acylation at lower temperatures. Much like LpxS induction, a decrease in hepta-acylation would increase membrane fluidity by reducing acyl chain packing within the OM. Another reason for reduced LpxM_Ab_ activity could arise from changes in the acyl-ACP pool. Bacteria synthesize unsaturated fatty acids at all temperatures using the FASII pathway (55). However, synthesis of unsaturated fatty acids is increased significantly during cold conditions for glycerophospholipid synthesis. Both LpxL_Ab_ and LpxM_Ab_ use lauroyl-ACP as the donor substrate, and perhaps the availability of C12:0 is decreased to fulfill the demand for unsaturated fatty acids at lower temperatures. Perhaps, A. baumannii not only upregulates LpxS expression but also downregulates *lpxM*_Ab_ transcription due to the double requirement of C12:0-ACP donor for lipid A synthesis.

The asymmetric lipid distribution of the OM along with the strong lateral interactions between LPS molecules contribute to an efficient barrier function against antimicrobial agents (2, 39). However, cold temperature alters OM permeability, resulting in antibiotic susceptibility including increased sensitivity to the normally excluded vancomycin glycopeptide (40, 56). In E. coli, LpxP activity is important for membrane permeability at 12°C as *lpxP* deletion increases sensitivity to vancomycin (56). A. baumannii resistance to vancomycin decreased significantly when grown at low temperatures (Fig. 7B). As expected, loss of a secondary acyl chain at the LpxL position increases antibiotic sensitivity regardless of growth temperature. However, at 37°C LpxL C12:0 addition resulted in a less permeable membrane whereas LpxS C8:0 addition promoted higher antibiotic resistance at 15°C (Fig. 7). Of course, it is possible that the differences in lipid A acylation influence other OM components, such as folding or insertion of OM β-barrels at different temperatures. Still, these data suggest that LpxS increases bacterial fitness under cold conditions.

It is unlikely that LpxS is active within a mammalian host, but we cannot discount that signals other than cold temperatures could induce expression. Still, since the number and length of lipid A acyl chains influence the inflammatory nature of LPS/LOS, we determined if LpxS could be useful in altering TLR4 activation. Like E. coli, A. baumannii lipid A species exhibit agonist activity for the TLR4/MD2 complex (Fig. 8) (23, 42), and substitution of a laurate at the 2′ position for an octanoate in either organism resulted in a reduced TLR4 response (Fig. 8). In Bordetella pertussis, a positive correlation is observed between shortening secondary acyl chain length (C16:0, C14:0, and C12:0) at the 2′ position and reduction of TLR4 activation (57). Also, synthetic hexa-acylated lipid A analogs carrying three secondary acyl chains of 10-carbon length exhibit optimal agonist activity whereas the 8-carbon length shows reduced TLR4 induction (58). Clearly, the length of the fatty acid at the 2′ position is relevant for TLR4 signaling. Given the use of lipid A variants in vaccine design (42, 59), the discovery of LpxS increases the dynamic range for the synthetic engineering of lipid A molecules to modulate the immune response.

Overall, little is known regarding how Gram-negative bacteria alter LPS structure under extreme growth conditions. The biological relevance of lipid A octanoylation in A. baumannii is likely to maintain membrane fluidity and permeability as a strategy for cold adaptation. LpxS acyltransferase exhibits selectivity for the shortest known acyl donor for a lipid A acyltransferase and shows positional specificity. Notably, this is a different approach than that of E. coli with incorporation of a long-chain unsaturated fatty acid by LpxP. Whether or not LpxS is induced under additional growth conditions to prevent membrane rigidification is unclear, but it seems likely that LpxS would support A. baumannii fitness in key environmental reservoirs.

## MATERIALS AND METHODS

### Bacterial and growth conditions.

All strains and plasmids used in this study are listed Table S3 in in the supplemental material. E. coli strains were grown in lysogeny broth (LB) or in low-phosphate M9 minimal medium (15.57 mM Na_2_HPO_4_, 8.81 mM KH_2_PO_4_, 8.55 mM NaCl, 18.69 mM NH_4_Cl, 0.1 mMCaCl_2_, 2 mM MgSO_4_, 0.4% glucose) supplemented with, 0.075 mM thiamine and 0.015 mM FeSO_4_, pH 7.0, at 30°C. Acinetobacter baumannii 17978, 19606, and 5075 strains were grown in LB at 15°C or 37°C. Antibiotics were used accordingly at the following concentrations: kanamycin at 10 μg/ml or 30 μg/ml, tetracycline at 10 μg/ml, and ampicillin at 100 μg/ml.

10.1128/mBio.01295-21.9 TABLE S3 Strains and plasmids used in this study. Download Table S3, PDF file, 0.06 MB. Copyright © 2021 Herrera et al. 2021 Herrera et al. https://creativecommons.org/licenses/by/4.0/ This content is distributed under the terms of the Creative Commons Attribution 4.0 International license.

### Recombinant DNA techniques.

Genomic DNA was extracted using the Easy-DNA genomic DNA (gDNA) purification kit (Invitrogen). Plasmid DNA was obtained with QIAprep Spin miniprep (Qiagen). PCRs were performed with TaKaRa *Ex Taq* DNA polymerase (TaKaRa). Custom synthetic oligonucleotides listed in Table S4 were manufactured by Integrated DNA Technologies (IDT). Amplified DNA products were separated in a 1% agarose gel and purified using the QIAquick gel extraction kit (Qiagen). DNA was concentrated with the DNA Clean and Concentrator kit (Zymo Research). For generation of recombinant plasmids, all restriction enzymes, T4 DNA ligase, and Antarctic phosphatase were purchased from New England BioLabs.

10.1128/mBio.01295-21.10 TABLE S4 Primers used in this study. Download Table S4, PDF file, 0.05 MB. Copyright © 2021 Herrera et al. 2021 Herrera et al. https://creativecommons.org/licenses/by/4.0/ This content is distributed under the terms of the Creative Commons Attribution 4.0 International license.

### Generation of deletion mutants.

The source of all E. coli late acyltransferase mutations, with the exception of *lpxL*, was from the Keio collection (60). Gene deletions were introduced via P1*vir* phage transduction, and candidate mutants were selected on 30 μg/ml kanamycin. Generation of the tetra-mutant strain (W3Δ4X) began with deletion of *lpxP* in strain W3110 at 37°C via transduction. The kanamycin resistance cassette was excised using the FLP-FLP recombination target (FRT) recombination system (61, 62) resulting in strain W3Δ*lpxP*. A similar procedure was used to introduce the *lpxM* mutation into strain W3Δ*lpxP*. To delete *lpxL*, P1*vir* phage from strain MLK53 (*lpxL*::Tn*10*) was used with selection at 30°C on M9 minimal agar medium (Difco) supplemented with 0.015 mM FeSO_4_, 0.075 mM thiamine, 5 mM sodium citrate, and tetracycline. The resulting triple mutant W3 Δ*lpxP* Δ*lpxM* Δ*lpxL* served as the host for the *pagP* mutation, yielding the tetra-acylated mutant W3Δ4X that was routinely grown at 30°C. All consecutive markerless deletions were verified by PCR using specific primers (Table S4).

Genome sequences for A. baumannii were obtained from the Prokaryotic Genome Analysis Tool Database (26). Plasmids and primers used are listed in Tables S3 and S4, respectively. Deletions in the 17978 strain were performed following the previously described REC_Ab_-one-step recombination system (63) using PCR products generated with either P1-P2 AblpxL primers for *lpxL* or P1-P2 A1S_1255 primers for *lpxS.* Mutants were selected on 10 μg/ml kanamycin and verified by PCR. To generate the Δ*lpxL*_Ab_ Δ*lpxS*_Ab_ double mutant, Δ*lpxL*_Ab_ served as the host strain for A1S_1255 deletion.

### Complementation plasmid constructs.

ORF *A1S_1255* from 17978 and ORF *lpxL* from E. coli were amplified by *Ex Taq* DNA polymerase using primers pMM_LpxS BamHI/EcoRI and pMM_EclpxL EcoRI/BamHI, respectively (Table S4). The resulting amplicons were cloned into the plasmid pMMB67EH, yielding plasmids pLpxS and pLpxL_Ec_. Plasmids were sequence verified using primers sqpMM-F/R (Table S4). Where appropriate, 0.05 mM IPTG (isopropyl-β-d-1-thiogalactopyranoside) was used to induce protein expression.

### Isolation and analysis of ^32^P-labeled lipid A.

E. coli and A. baumannii cells were labeled using 2.5 and 5 μCi/ml ^32^P_i_ (Perkin-Elmer), respectively. The lipid domain of LPS/LOS was released via mild-acid hydrolysis (pH 4.5, 100°C, 30 min) that cleaves the linkage between the lipid A and the first Kdo sugar of the core oligosaccharide. Lipid A species were extracted as previously described (38, 64) and separated by thin-layer chromatography (TLC) in a solvent system of chloroform, pyridine, 88% formic acid, and water (50:50:16:5, vol/vol, respectively). Radiolabeled lipids were visualized by phosphorimaging analysis.

### Mass spectrometry.

Lipid A for MALDI-TOF mass spectrometry analysis was extracted as previously described (64) and dissolved in chloroform-methanol (4:1, vol/vol). The matrix 5-chloro-mercaptobenzothiazole (CMBT) (Sigma-Aldrich) was dissolved in chloroform-methanol-water (4:4:1, vol/vol/vol) at a concentration of 20 mg/ml (65). A matrix mixture was then prepared by combining CMBT with the additive saturated tribasic ammonium sulfate (20:1, vol/vol). MALDI plates were spotted with the sample and matrix mixture in a 1:1 (vol/vol) ratio, and spectra were acquired using negative ion reflectron mode on an Autoflex Speed mass spectrometer (Bruker Daltonics). A total of 500 single laser shots were averaged from each mass spectrum. Data were processed using FlexControl 3.4 and FlexAnalysis 3.4 software (Bruker Daltonics).

### RNA isolation and quantitative real-time PCR.

Overnight cultures were diluted 1:100 in LB, incubated at 37°C or at 15°C, and grown to an optical density at 600 nm (OD_600_) of ∼0.5 to 0.6. One volume of bacterial culture and 2 volumes of RNAprotect bacterial reagent (Qiagen) were mixed by vortexing and incubated for 5 min at room temperature. Cells were harvested by centrifugation at 5,000 × *g* for 10 min at room temperature. RNA was isolated using the SV total RNA isolation system (Promega). To complete removal of DNA, total RNA was treated with RQ1 RNase-free DNase (Promega). First-strand cDNA was generated using a high-capacity cDNA reverse transcription kit (AB Applied Biosystems). Transcripts were quantified by qPCR using SYBR green premix (AB Applied Biosystems) and specific primers listed in Table S4. Results were analyzed using the LightCycler 96 System (Roche). Relative expression was calculated as the ratio of the target gene in comparison to the internal standard *gyrA* gene using the Pfaffl method (66).

### HEK-Blue hTLR4 assay.

HEK-Blue hTLR4 and HEK-Blue Null2 cells (InvivoGen) were grown using the manufacturer’s specifications. For hTLR4 stimulation, bacteria were grown in LB with appropriate antibiotics for plasmid-containing strains. For strains with complemented *lpxL*_Ec_ or *lpxS*_Ab_ in *trans*, IPTG was added to a final concentration of 0.05 mM. Bacteria were grown until mid-log and normalized to an OD_600_ of 1.0, pelleted at 17,000 × *g* for 2 min, and resuspended in 1 ml sterile phosphate-buffered saline (PBS). The wash step was repeated once more before the bacteria were heat killed by boiling for 20 min in 1 ml PBS.

Commercially available TLR agonist (E. coli LPS) and antagonist (Rhodobacter sphaeroides LPS) were prepared using manufacturer’s recommendations (InvivoGen) and used as controls to ensure appropriate HEK-Blue reporter activity (see Fig. S6C). Serial dilutions of heat-killed bacteria and LPS were made in 96-well plates with sterile PBS. HEK-Blue tissue culture cells were washed and resuspended in PBS. Cells were diluted to a concentration of 140,000 cells/ml in HEK-Blue detection medium (InvivoGen). One hundred eighty microliters of the cell suspension was aliquoted to each well of a 96-well plate and stimulated using 20 μl of bacterial dilutions overnight. SEAP production was determined by reading absorbance at 637 nm. Values were analyzed by analysis of variance (ANOVA) with Sidak’s multiple test correction performed with GraphPad Prism (San Diego, CA).

### Multiple sequence alignment.

Amino acid and 16S sequences were obtained from BioCyc (67). Sequences were aligned, and the phylogenetic tree data were generated using Clustal Omega (68). Phylogenetic trees were generated in R with RStudio IDE using the TreeTools package.

### MIC determinations.

For vancomycin MIC determination by E-strips (69), overnight cultures were diluted 1:100 into fresh LB medium and grown to an OD_600_ of 0.5 at the indicated temperature. Following a 1:10 dilution, the cells were plated immediately on LB agar containing proper antibiotic and 0.05 mM IPTG where necessary. A vancomycin Etest gradient strip (bioMérieux) was applied onto inoculated plates and evaluated after overnight incubation at 37°C or after 2 days at 15°C.

As novobiocin E-strips were not available, the novobiocin MIC was determined by antibiotic serial dilutions as previously described (70). Briefly, LB containing 64 μg/ml novobiocin followed 2-fold serial dilutions. Overnight cultures were diluted and aliquoted to 1:1,000 dilution in 4 ml. Cultures were incubated at 37°C for 16 h or at 15°C for 40 h under shaking conditions, and growth was monitored by OD_600_. Under both conditions, untreated controls reached similar growth. The MIC was defined as 90% of growth inhibition relative to the untreated control.
