# Supplementary material for: Homeoviscous Adaptation of the Acinetobacter baumannii Outer Membrane: Alteration of Lipooligosaccharide Structure during Cold Stress
Source: mBio. 2021 Aug 24;12(4):e01295-21. doi: 10.1128/mBio.01295-21 (PMC8406137; doi:10.1128/mBio.01295-21)
Supplement: FIG S1 [file mbio.01295-21-sf001.pdf]

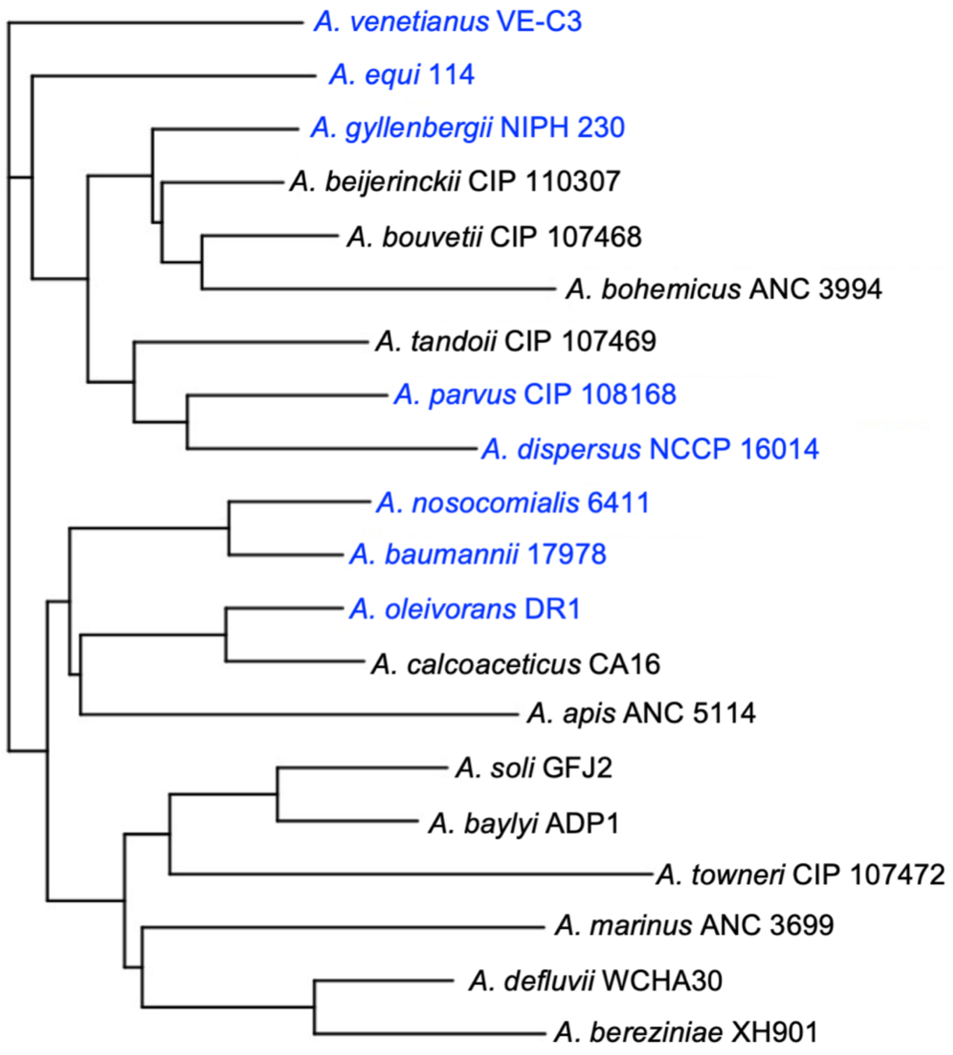

Fig S1: LpxS lipid A acyltransferase is distributed within *Acinetobacter* species. Phylogenetic distribution of selected *Acinetobacter* species is based on 16S rRNA. *Acinetobacter* strains that contain a single copy of *lpxS* in their genomes are indicated in blue color.
