# Supplementary material for: Homeoviscous Adaptation of the Acinetobacter baumannii Outer Membrane: Alteration of Lipooligosaccharide Structure during Cold Stress
Source: mBio. 2021 Aug 24;12(4):e01295-21. doi: 10.1128/mBio.01295-21 (PMC8406137; doi:10.1128/mBio.01295-21)
Supplement: TABLE S1 [file mbio.01295-21-st001.pdf]

Table S1. Distribution of putative lipid A secondary acyltransferases in *Acinetobacter*

Organism[a]	Gene	BLASTp[b] LpxL	BLASTp[b] LpxS	BLASTp[b] LpxM	Prediction[c]
Acinetobacter apis	CFY84_RS06810	80,90,0, E = 0.0	63,74,1, E = 6e-138	26,44,5, E = 3e-21	LpxL
ANC 5114	CFY84_RS04375	25,45,4, E = 2e-19	22,43,4, E = 2e-17	63,81,0, E = 2e-150	LpxM
Acinetobacter baylyi ADP1	ACIAD_RS02200	89,95,0, E = 0.0	65,78,1, E = 7e-154	27,44,6, E = 1e-17	LpxL
	ACIAD_RS12000	24,44,7, E = 2e-18	23,43,4, E = 1e-16	75,85,0, E = 6e-178	LpxM
Acinetobacter beijerinckii	F933_RS11345	80,91,0, E = 0.0	64,77,2, E = 2e-149	25,43,4, E = 2e-19	LpxL
CIP 110307	F933_RS13970	68,84,1, E = 2e-167	65,81,0, E = 7e-158	27,43,6, E = 3e-14	LpxS
	F933_RS15275	27,47,6, E= 4e-21	23,44,6, E = 3e-14	69,82,0, E = 3e-158	LpxM
	F933_RS11985	23,45,6, E = 8e-19	25,44,8, E = 1e-13	35,57,1, E = 1e-56	LpxM-2
Acinetobacter bereziniae	BSR55_RS18775	83,92,0, E = 0.0	66,77,1, E = 5e-150	26,44,4, E = 3e-18	LpxL
XH901	BSR55_RS03900	25,43,3, E = 7e-20	23,42,8, E = 2e-12	38,56,2, E = 1e-61	LpxM-2
	BSR55_RS07480	25,44,7, E = 2e-18	22,42,5, E = 1e-13	64,80,0, E = 3e-152	LpxM
Acinetobacter bohemicus	F994_RS08490	78,89,0, E = 0.0	65,77,1, E = 6e-150	27,45,4, E = 7e-21	LpxL
ANC 3994	F994_RS09715	25,45,5, E = 7e-21	22,42,5, E = 2e-12	38,57,1, E = 1e-63	LpxM-2
	F994_RS01865	27,45,5, E = 7e-20	27,42,5, E = 7e-18	68,83,0, E = 1e-161	LpxM
Acinetobacter bouvetii	F941_RS02110	77,88,0, E = 0.0	65,77,1, E = 1e-149	26,43,4, E = 5e-17	LpxL
CIP 107468	F941_RS05230	29,47,5, E = 9e-25	24,45,5, E = 4e-19	69,84,0, E = 2e-165	LpxM
Acinetobacter calcoaceticus	BUM88_RS02120	94,98,0, E = 0.0	65,79,1, E = 4e-148	27,43,4, E = 1e-17	LpxL
CA16	BUM88_RS05895	68,81,1, E = 5e-160	83,90,0, E = 0.0	30,45,7, E = 7e-20	LpxS
	BUM88_RS15045	27,46,7, E = 7e-20	22,43,4, E = 8e-16	86,96,0, E = 0.0	LpxM
Acinetobacter defluvii	DJ533_RS16670	85,93,0, E = 0.0	66,76,1, E = 2e-150	26,42,3, E = 1e-18	LpxL
WCHA30	DJ533_RS07535	24,46,4, E = 3e-18	22,41,4, E = 4e-16	69,84,0, E = 4e-165	LpxM
Acinetobacter disperses	FPL17_RS11850	85,92,0, E = 0.0	66,78,1, E = 2e-152	27,43,4, E = 2e-18	LpxL
NCCP 16014	FPL17_RS05100	70,86,1, E = 1e-173	68,81,0, E = 3e-162	26,43,5, E = 1e-17	LpxS
	FPL17_RS17455	25,46,3, E = 3e-21	25,44,7, E = 6e-14	36,58,1, E = 3e-63	LpxM-2
	FPL17_RS08305	25,46,5, E = 4e-19	23,44,7, E = 1e-13	68,84,0, E = 1e-164	LpxM
Acinetobacter equi 114	AOY20_RS05835	80,89,0, E = 0.0)	63,75,1, E = 9e-146	27,44,4, E = 8e-21	LpxL
	AOY20_RS09275	64,79,1, E = 4e-144	63,76,0, E = 1e-136	24,43,5, E = 2e-13	LpxS
	AOY20_RS06495	22,42,5, E = 1e-18	24,42,3, E = 7e-17	37,57,1, E = 7e-63	LpxM-2
	AOY20_RS13950	22,41,4, E = 1e-11	22,39,9, E = 8e-9	61,75,0, E = 3e-137	LpxM
Acinetobacter gyllenbergii	F987_RS08610	86,94,0, E = 0.0	66,78,1, E = 7e-152	27,43,4, E = 7e-20	LpxL
NIPH 230	F987_RS19635	71,85,1, E = 9e-172	66,80,0, E = 1e-158	27,41,5, E = 3e-15	LpxS
	F987_RS02475	24,45,6, E = 3e-19	25,46,5, E = 5e-14	36,59,1, E = 3e-60	LpxM-2
	F987_RS13610	25,47,6, E = 5e-19	23,44,7, E = 1e-15	69,85,0, E = 6e-168	LpxM
Acinetobacter marinus	BLQ60_RS12235	72,86,0, E = 1e-167	56,75,1, E = 2e-127	26,42,3, E = 2e-16	LpxL
ANC 3699	BLQ60_RS04425	27,46,8, E = 4e-22	25,43,8, E = 6e-17	38,58,1, E = 1e-63	LpxM-2
	BLQ60_RS09420	25,44,4, E = 8e-19	24,42,4, E = 3e-14	52,71,0, E = 6e-109	LpxM
Acinetobacter nosocomailis	FDQ49_RS07345	98,99,0, E = 0.0	67,78,1, E = 1e-155	28,44,5, E = 3e-17	LpxL
6411	FDQ49_RS15770	65,78,1, E = 5e-154	95,97,0, E = 0.0	27,44,4, E = 2e-19	LpxS
	FDQ49_RS13960	28,46,5, E = 2e-20	25,44,4, E = 8e-19	91,97,0, E = 0.0	LpxM
Acinetobacter oleivorans	AOLE_RS17285	96,99,0, E = 0.0	65,79,1, E = 6e-149	28,44,5, E = 1e-17	LpxL
DR1	AOLE_RS13210	67,80,1, E = 5e-158	84,91,0, E = 0.0	28,44,5, E = 9e-21	LpxS
	AOLE_RS04400	28,47,5, E = 5e-21	24,44,4, E = 1e-16	88,96,0, E = 0.0	LpxM
Acinetobacter parvus	F988_RS00910	80,90,0, E = 0.0	64,76,1, E = 7e-147	27,44,4, E = 1e-18	LpxL
CIP 108168	F988_RS11410	25,45,6, E = 8e-22	26,45,5, E = 9e-17	37,58,1, E = 8e-64	LpxM-2
	F988_RS09405	25,46,5, E = 7e-19	22,43,7, E = 2e-15	67,83,0, E = 2e-163	LpxM
Acinetobacter soli GFJ2	BEN76_RS15395	86,95,0, E = 0.0	65,79,1, E = 2e-155	26,42,4, E = 8e-18	LpxL
	BEN76_RS01355	24,45,4, E= 5e-19	23,43,4, E = 8e-17	75,85,0, E = 1e-177	LpxM
Acinetobacter tandoii	I593_RS14195	87,92,0, E = 0.0	66,78,1, E = 3e-154	31,46,5, E = 8e-24	LpxL
CIP 107469	I593_RS02060	27,44,3, E = 3e-21	25,44,4, E = 2e-19	70,82,0, E = 3e-163	LpxM
	I593_RS13775	23,45,5, E = 2e-16	19,42,3, E = 2e-10	36,55,4, E = 2e-56	LpxM-2
Acinetobacter towneri	F947_RS12185	78,86,0, E = 0.0	63,74,1, E = 4e-146	27,44,6, E = 7e-18	LpxL
CIP 107472	F947_RS02650	27,47,4, E = 2e-23	26,46,5, E = 7e-21	65,80,0, E = 1e-151	LpxM
	F947_RS09645	21,44,2, E = 7e-18	19,44,3, E = 2e-12	36,59,2, E = 1e-61	LpxM-2
Acinetobacter venetianus	A489_RS0115890	84,92,0, E = 0.0	67,77,1, E = 4e-153	27,43,4, E = 6e-20	LpxL
VE-C3	A489_RS0108770	65,84,1, E = 5e-158	61,78,0, E = 5e-151	24,42,4, E = 1e-14	LpxS
	A489_RS0101420	25,45,3, E = 6e-22	25,45,8, E = 7e-14	34,59,1, E = 5e-57	LpxM-2
	A489_RS0103505	26,45,8, E = 2e-19	23,43,8, E = 3e-14	69,83,0, E = 4e-166	LpxM

[a]Names in blue indicate strains with assigned LpxS homolog

[b]Numbers indicate BLASTp percentage identity, positive, gaps compared to 17978

[c]Predicted acyltransferase homolog. LpxM-2 indicates a secondary LpxM ortholog
