# Supplementary material for: Homeoviscous Adaptation of the Acinetobacter baumannii Outer Membrane: Alteration of Lipooligosaccharide Structure during Cold Stress
Source: mBio. 2021 Aug 24;12(4):e01295-21. doi: 10.1128/mBio.01295-21 (PMC8406137; doi:10.1128/mBio.01295-21)
Supplement: FIG S3 [file mbio.01295-21-sf003.pdf]

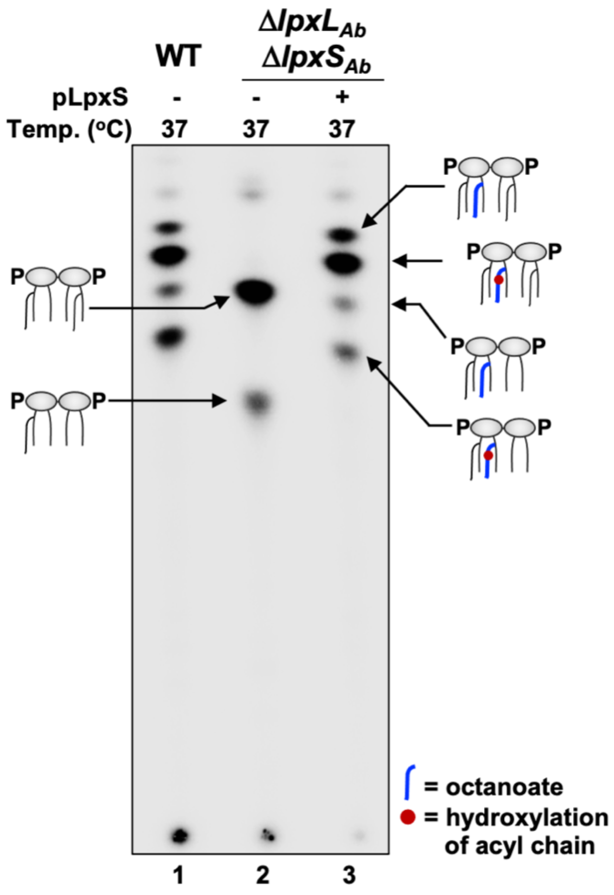

FIG S3: Lipid A profile of the Δ*lpxL*Ab, Δ*lpxS*Ab double mutant grown in LB medium at 37°C. The double mutant exhibits a similar lipid A phenotype at 37°C (lane 2) and at 15°C (Fig. 5B, lane 2). LpxS overexpression restores lipid A acylation similar to WT (lane 1 and 3). Lipid A samples were separated by TLC. Lipid A species are depicted as cartoons with octanoate in blue and hydroxylation by LpxO in red.
