# Supplementary material for: Homeoviscous Adaptation of the Acinetobacter baumannii Outer Membrane: Alteration of Lipooligosaccharide Structure during Cold Stress
Source: mBio. 2021 Aug 24;12(4):e01295-21. doi: 10.1128/mBio.01295-21 (PMC8406137; doi:10.1128/mBio.01295-21)
Supplement: FIG S4 [file mbio.01295-21-sf004.pdf]

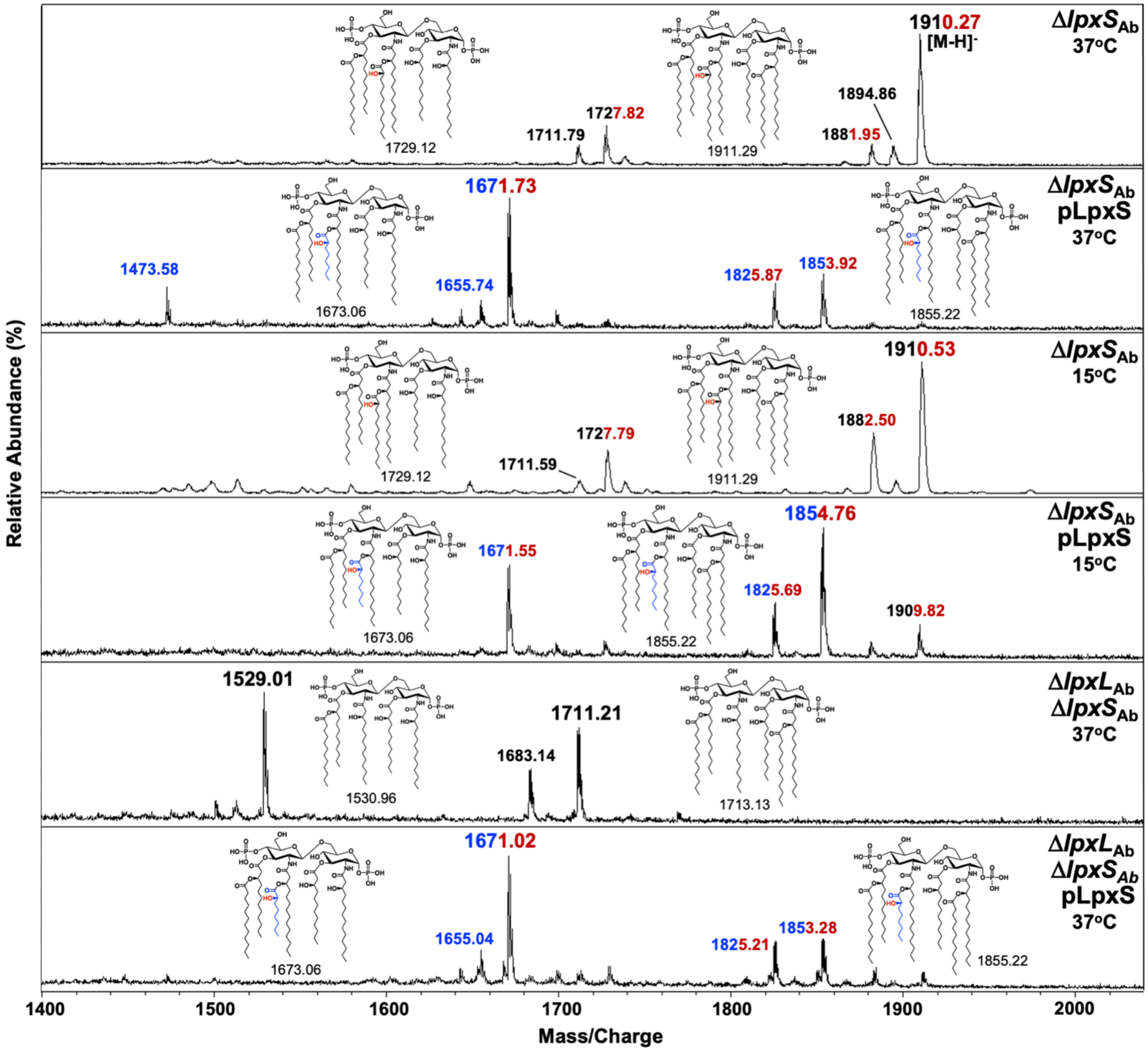

FIG S4: MALDI-TOF MS from Δ*lpxS*Ab complemented strain and Δ*lpxL*Ab Δ*lpxS*Ab double mutant overexpressing LpxS were evaluated and compared to their respective control strains. Numbers in blue color show octanoylated lipid A whereas red color indicates hydroxylated acyl chain at the position 2'.
