# Supplementary material for: Homeoviscous Adaptation of the Acinetobacter baumannii Outer Membrane: Alteration of Lipooligosaccharide Structure during Cold Stress
Source: mBio. 2021 Aug 24;12(4):e01295-21. doi: 10.1128/mBio.01295-21 (PMC8406137; doi:10.1128/mBio.01295-21)
Supplement: FIG S5 [file mbio.01295-21-sf005.pdf]

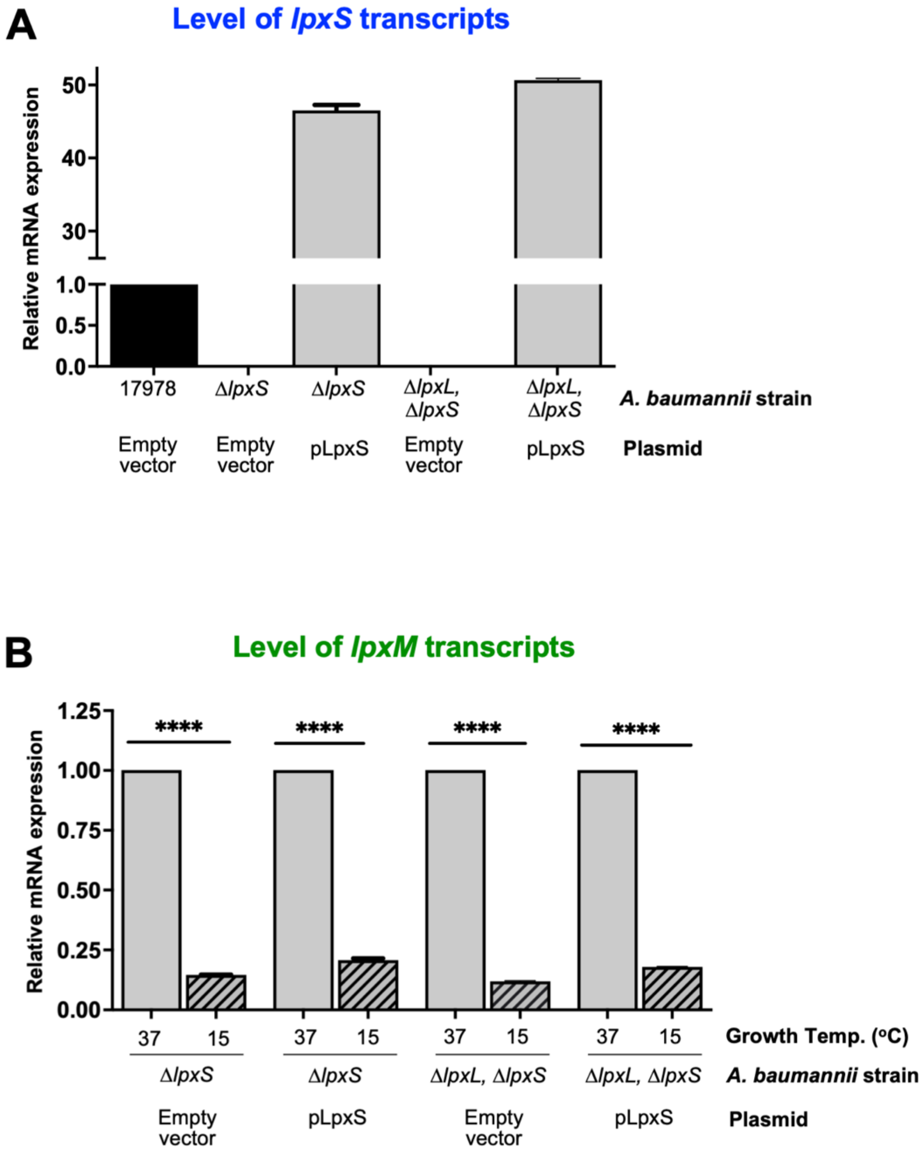

FIG S5: Controls for RT-qPCR analysis of acyltransferase gene expression. **(A)** Controls for mutant and complemented strains. *lpxS* transcript levels determined in Δ*lpxS*$_{Ab}$ and the Δ*lpxL*$_{Ab}$, Δ*lpxS*$_{Ab}$ double mutant carrying empty plasmid or pLpxS relative to WT 17978. Cells were grown in LB medium at 15°C. **(B)** *lpxM* expression is downregulated in response to cold temperature independently of other acyltransferase gene deletions. Δ*lpxS*$_{Ab}$ and Δ*lpxL*$_{Ab}$, Δ*lpxS*$_{Ab}$ carrying empty plasmid or overexpressing LpxS were grown in LB at 37°C and 15°C. Ratio of *lpxM* transcript levels at 15°C is relative to 37°C. Level of transcripts are normalized using *gyrA* as the reference gene. Data represent the average from three biological repetitions. Statistical significance (****) was calculated using *t*-test ($P < 0.001$).
