# Supplementary material for: Homeoviscous Adaptation of the Acinetobacter baumannii Outer Membrane: Alteration of Lipooligosaccharide Structure during Cold Stress
Source: mBio. 2021 Aug 24;12(4):e01295-21. doi: 10.1128/mBio.01295-21 (PMC8406137; doi:10.1128/mBio.01295-21)
Supplement: TABLE S2 [file mbio.01295-21-st002.pdf]

Table S2: Novobiocin minimal inhibitory concentration (MIC)

Strains/temperature	37ºC	15ºC
17978 p	6.0 ± 1.0	1.5 ± 0.25
Δ*lpxL* Δ*lpxS* p	2.0 ± 0.5	1.0± 0
Δ*lpxL* Δ*lpxS* pLpxL	6.0 ± 1.0	1.0± 0
Δ*lpxL* Δ*lpxS* pLpxS	3.0 ± 0.5	1.5 ± 0.25

Data represent mean of three biological repetitions and standard deviation. p: empty pMMB67EH
