# Supplementary material for: Homeoviscous Adaptation of the Acinetobacter baumannii Outer Membrane: Alteration of Lipooligosaccharide Structure during Cold Stress
Source: mBio. 2021 Aug 24;12(4):e01295-21. doi: 10.1128/mBio.01295-21 (PMC8406137; doi:10.1128/mBio.01295-21)
Supplement: FIG S6 [file mbio.01295-21-sf006.pdf]

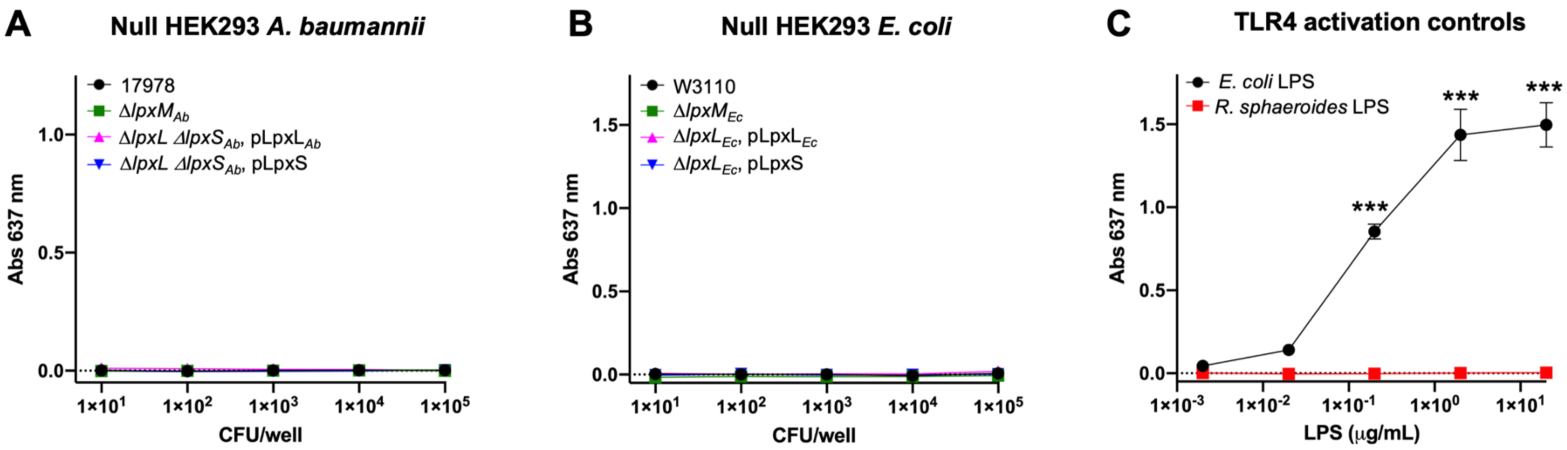

FIG S6: Controls for TLR4 Assays. Cells from **(A)** WT 17978, Δ*lpxM*Ab, Δ*lpxL*Ab Δ*lpxS*Ab overexpressing LpxSAb or LpxLAb, and **(B)** WT W3110, Δ*lpxM*Ec, Δ*lpxL*Ec pLpxLEc, Δ*lpxL*Ec pLpxS showed no induction on null HEK293 cells that served as negative control for TLR4. **(C)** Purified LPS from *E. coli* (agonist) and *R. sphaeroides* (antagonist) were evaluated at different concentrations for TLR4 stimulation in the reporter HEK-Blue hTLR4 cells.
