# Supplementary material for: Homeoviscous Adaptation of the Acinetobacter baumannii Outer Membrane: Alteration of Lipooligosaccharide Structure during Cold Stress
Source: mBio. 2021 Aug 24;12(4):e01295-21. doi: 10.1128/mBio.01295-21 (PMC8406137; doi:10.1128/mBio.01295-21)
Supplement: TABLE S3 [file mbio.01295-21-st003.pdf]

Table S3. Strains and plasmids used in this study

Strains or plasmids	Genotype or description	Reference
E. coli		
XL1 blue	*recA1 endA1 gyrA96 thi-1 hsdR17 supE44 relA1 lac* [F´ *proAB lacI*^{q}*Z*Δ*M15* Tn*10* (Tet^{R})]	Stratagene
W3110	Wild type, F-, *λ*$^{-}$, IN(*rrnD*, *rrnE*)1, *rph*-1	Coli Genetic Stock Center (Yale)
Δ*lpxL*$_{Ec}$	MLK53, F-, *λ*$^{-}$, *lpxL10*(ts)*::mini-Tn10*, *IN(rrnD-rrnE)1*, *rph-1,* Tet^{R}	Coli Genetic Stock Center (Yale)
Δ*lpxM*$_{Ec}$	W3110, Δ*lpxM*	This study
W3Δ4X	W3110, Δ*lpxP*, Δ*lpxM* Δ*lpxL* Δ*pagP*, Tet^{R}	This Study
A. baumannii		
17978	Wild type	ATCC
19606	Wild type	ATCC
5075	Wild type	ATCC
Δ*lpxL*$_{ab}$	17978, Δ*lpxL* (A1S_0431)	This study
Δ*lpxM*$_{Ab}$	17978, Δ*lpxM*	(1)
Δ*lpxS*$_{Ab}$	17978, ΔA1S_1255	This study
Δ*lpxL*$_{Ab}$ Δ*lpxS*$_{Ab}$	17978, Δ*lpxL* ΔA1S_1255	This study
**Plasmids**		
pMMB67EH	Expression vector, Kan^{R}	(2)
pREC$_{Ab}$	pMMB67EH:REC$_{Ab,}$ Tet^{R}	(3)
pFLP	pMMB67EH:FLP, Tet^{R}	(3)
pLpxS	pMMB67EH::A1S_1255, Kan^{R}	This study
pLpxL$_{Ab}$	pMMB67EH::LpxL$_{Ab}$, Amp^{R}	(1)
pLpxL$_{Ec}$	pMMB67EH::LpxL$_{Ec,,}$ Kan^{R}	This study

Tet^{R}: tetracycline, Kan^{R}: kanamycin and Amp^{R}: ampicillin, resistant.

References

1. Boll JM, Tucker AT, Klein DR, Beltran AM, Brodbelt JS, Davies BW, Trent MS. 2015. Reinforcing lipid A acylation on the cell surface of *Acinetobacter baumannii* promotes cationic antimicrobial peptide resistance and desiccation survival. mBio 6:e00478-15.

2. Boll JM, Crofts AA, Peters K, Cattoir V, Vollmer W, Davies BW, Trent MS. 2016. A penicillin-binding protein inhibits selection of colistin-resistant, lipooligosaccharide-deficient *Acinetobacter baumannii*. Proc Natl Acad Sci 113:E6228–E6237.
3. Tucker AT, Nowicki EM, Boll JM, Knauf GA, Burdis NC, Trent MS, Davies BW. 2014. Defining gene-phenotype relationships in *Acinetobacter baumannii* through one-step chromosomal gene inactivation. mBio 5:e01313-14.
