# Supplementary material for: Homeoviscous Adaptation of the Acinetobacter baumannii Outer Membrane: Alteration of Lipooligosaccharide Structure during Cold Stress
Source: mBio. 2021 Aug 24;12(4):e01295-21. doi: 10.1128/mBio.01295-21 (PMC8406137; doi:10.1128/mBio.01295-21)
Supplement: TABLE S4 [file mbio.01295-21-st004.pdf]

Table S4. Primers used in this study

Name	Sequence (5'-3')	Purpose
lpxP F	CACTCTTTACAGGAACCATTGTCGTACATG	Verification deletion *lpxP E. coli*
lpxP R	GTCTCATATCGGCGACAGTTTAATTTCTGAC	Verification deletion *lpxP E. coli*
lpxM F	CGCAGCCGGTACGCAGTCAG	Verification deletion *lpxM E. coli*
lpxM R	CCGGCCTACAGTTCAATGATAGTTCAAC	Verification deletion *lpxM E. coli*
lpxL F	GCGGCAATTTCGCCCAGTCTTCAG	Verification deletion *lpxL E. coli*
lpxL R	GACTTCCAGGGGGCGCAATCC	Verification deletion *lpxL E. coli*
PagP F	CTTTTTTGACTATTCCCATCGC	Verification deletion *pagP E. coli*
PagP R	ATTGTGGTACGCTTTGTCCA	Verification deletion *pagP E. coli*
P1-AblpxL	TAACCCGCTTTATTTTTCTTTATCGCGTCTAAACCTTCAATTATACTCCTGT GTATTACGACGATAAATTCAGTTCGTGATACACTTTTGGCAGTTTTAACTT TTTAAATTCTTTATATCGTT ATGAGC GTGTAGGCTGGAGCTGCTTC	Deletion *lpxL A. baumannii*
P2-AblpxL	CAAAGGATTTTTGCTTACTTTTTATCCCTAAAAAGTAAGGTCTGCCTATCC GAAAGTATTTGAATATCACTTTAAATCGAGGCTGTGCCTATTACTAATCTA AAATTATTACAGCCTTATCTTAATAAAT CATATGAATATCCTCCTTAG	Deletion *lpxL A. baumannii*
P1 A1S_1255	AAGAATAAATCGGAGCACTCACCAGTACTGTTTAGTAGCTGTTCGGTAGA TTGAGCCTAATATTAAGACACACTTTACAAACGTATGTAGAGTGCGAGTGT GTTAGGATGAATTTCTATACCTCTAGATATTTACAGTATGACGAAACCTGT GTAGGCTGGAGCTGCTTC	Deletion A1S_1255 *A. baumannii*
P2 A1S_1255	TGACTTTTGTTTTAAATTAGATCATTAAAAACTAAGGATAAATAATTAAAAA TTATTGTTAATTTAGGATTTATAAAGTCTCGGTTTAAAGGCCTTTAAATCTC TCAGTTGACTGTATAAAAAGGCATAACAAAATCAGTAAAACTTCGCCATAT GAATATCCTCCTTAG	Deletion A1S_1255 *A. baumannii*
ver_1255F	TGATCGCAGCTTTGTACAGG	Verification deletion *A1S_1255*
ver_1255R	TAGCCAAGCCCCATACAGTC	Verification deletion *A1S_1255*
Ver_lpxLAb	CTAGAACACCGCCGATAACC	Verification deletion *lpxL*$_{Ab}$
Ver_lpxLAb	TGTAACATCAGCGGAACAAAG	Verification deletion *lpxL*$_{Ab}$
pMM_LpxSEcoRI	ATTTAACCGGAATTCATGACGAAACCTGAAGCGCAG	Cloning *A1S_1255* into pMMB67EH
pMM_LpxSBamHI	TAATAGCGGATCCTCAGTAAAACTTCGCTTCACCAG	Cloning *A1S_1255* into pMMB67EH
pMM_EcLpxLEcoRI	ATTTAACCGGAATTCATGACGAATCTACCCAAGTTCTCC	Cloning *lpxL E.coli* into pMMB67EH
pMM_EcLpxL BamHI	TAATAGCGGATCCTTAATAGCGTGAAGGAACGCCTTCC	Cloning *lpxL E.coli* into pMMB67EH
sqpMMF	CGGTTCTGGCAAATATTCTG	Sequencing primer for pMMB67EH constructs.
sqpMMR	TGCCGCCAGGCAAATTCTG	Sequencing primer for pMMB67EH constructs

qlpxL F	CCCTGAATGGTCACCTGAAA	Quantification *lpxL* transcript *A.baumannii*
qlpxL R	ACCTTTGCCTTGAGCTTGTG	Quantification *lpxL* transcript *A.baumannii*
qlpxM F	AAGCGAATCTCGTCCCTACC	Quantification *lpxM* transcript *A.baumannii*
qlpxM R	CTTGAGGCAAGTGGAACACC	Quantification *lpxM* transcript *A.baumannii*
qlpxS F	CGAATGCGAAGTGAATGATG	Quantification *lpxS* transcript *A.baumannii*
qlpxS R	CTTCACCAGCAGGACGAGTT	Quantification *lpxS* transcript *A.baumannii*
qgyrA F	TGACTTCCCGACCAAGAAAC	Quantification *gyrA* transcript *A.baumannii*
qgyrA R	TACGAACAAGCGTACCACCA	Quantification *gyrA* transcript *A.baumannii*
