# Supplementary material for: Homeoviscous Adaptation of the Acinetobacter baumannii Outer Membrane: Alteration of Lipooligosaccharide Structure during Cold Stress
Source: mBio. 2021 Aug 24;12(4):e01295-21. doi: 10.1128/mBio.01295-21 (PMC8406137; doi:10.1128/mBio.01295-21)
Supplement: FIG S2 [file mbio.01295-21-sf002.pdf]

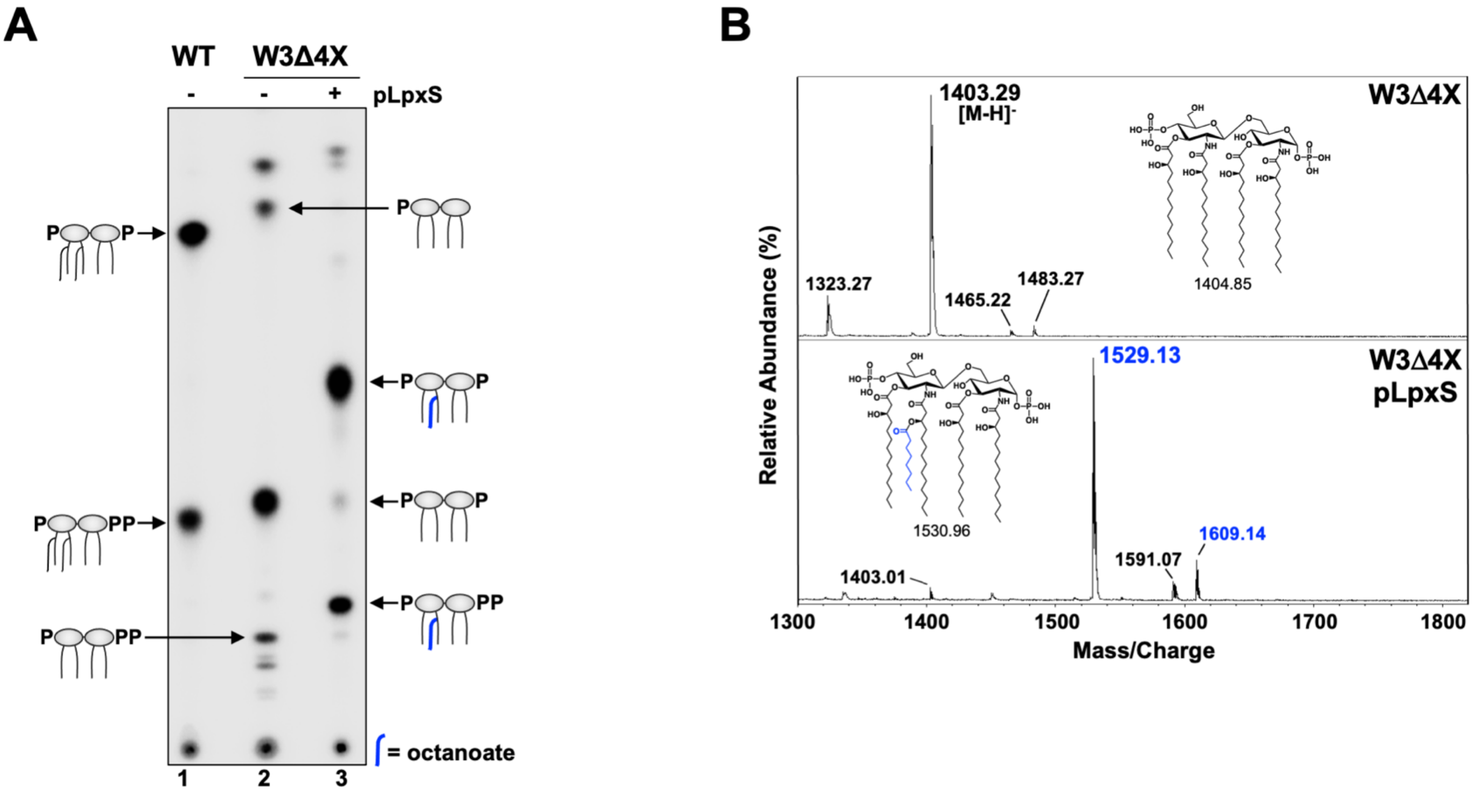

FIG S2: LpxS attaches a single secondary acyl chain. (A) TLC analysis of ^{32}P-labeled lipid A from the *E. coli* strain W3Δ*lpxL*, Δ*lpxM*, Δ*lpxP*, Δ*pagP* (W3Δ4X) tetra-acylated mutant expressing LpxS. Cells were grown in the presence of $^{32}P_i$ at 30°C followed by lipid A extraction. Lipid A species are depicted as cartoons, with the octanoate fatty acid colored blue. (B) MALDI-TOF mass spectrometry of lipid A from W3Δ4X with or without LpxS expression. Lipid A samples were analyzed in the reflectron negative mode and indicate LpxS-dependent octanoate addition. Octanoylated lipid A species are indicated in blue color and chemical structures and exact masses are shown.
